# Multifunctional Electrospun Materials from Poly(Vinyl Alcohol)/Chitosan and Polylactide Incorporating Rosmarinic Acid and Lidocaine with Antioxidant and Antimicrobial Properties

**DOI:** 10.3390/polym17192657

**Published:** 2025-09-30

**Authors:** Milena Ignatova, Dilyana Paneva, Selin Kyuchyuk, Nevena Manolova, Iliya Rashkov, Milena Mourdjeva, Nadya Markova

**Affiliations:** 1 Laboratory of Bioactive Polymers, Institute of Polymers, Bulgarian Academy of Sciences, Acad. G. Bonchev St., Bl. 103A, 1113 Sofia, Bulgaria; panevad@polymer.bas.bg (D.P.); selin.erdinch@polymer.bas.bg (S.K.); manolova@polymer.bas.bg (N.M.); 2 Institute of Biology and Immunology of Reproduction “Acad. Kiril Bratanov”, Bulgarian Academy of Sciences, 73, Tsarigradsko Shose Blvd., 1113 Sofia, Bulgaria; mourdjeva@ibir.bas.bg; 3 Institute of Microbiology, Bulgarian Academy of Sciences, Acad. G. Bonchev St., Bl. 26, 1113 Sofia, Bulgaria; nadya.markova@microbio.bas.bg

**Keywords:** multifunctional materials, chitosan, poly(vinyl alcohol), poly(L-lactide), dual spinneret electrospinning, rosmarinic acid, lidocaine hydrochloride, antioxidant, antimicrobial properties

## Abstract

Novel multifunctional fibrous materials were prepared by simultaneous dual spinneret electrospinning of two separate solutions differing in composition. This technique allowed for the preparation of materials built of two types of fibers: fibers from poly(vinyl alcohol) (PVA), chitosan (Ch), and rosmarinic acid (RA), and poly(L-lactide) (PLA) fibers containing lidocaine hydrochloride (LHC). Confocal laser scanning microscopy (CLSM) analyses showed that both types of fibers are present on the surface and in the bulk of the new materials. The presence of all components and some interactions between them were proven by attenuated total reflectance Fourier transform infrared (ATR-FTIR) spectroscopy. RA and LHC were in an amorphous state in the fibers, and their presence affected the temperature characteristics and the crystallinity, as detected by differential scanning calorimetry (DSC) and X-ray diffraction analyses (XRD). The presence of PVA/Ch/RA fibers enabled the hydrophilization of the surface of the multifunctional fibrous materials (the water contact angle value was 0°). The newly developed materials demonstrated adequate mechanical properties, making them suitable for use in wound dressing applications. The RA-containing fibrous mats possessed high radical-scavenging activity (ca. 93%), and the combining with LHC led to an enhancement of this effect (ca. 98.5%). RA-containing fibrous mats killed all the pathogenic bacteria *S. aureus* and *E. coli* and decreased the titer of fungi *C. albicans* by ca. 0.4 log for a contact time of 24 h. Therefore, the new materials are prospective as antibacterial and atraumatic functional wound dressings, as systems for local drug delivery, and in medical skincare.

## 1. Introduction

Electrospinning has emerged as a highly promising technology for the facile and efficient production of multifunctional fibrous materials [1,2]. Regarding biomedical applications, fibrous materials loaded with bioactive compounds attract significant attention due to the complexity of the beneficial properties that might be imparted to them (adequate hydrophilicity and mechanical behavior, antioxidant and antimicrobial activity). The hydrophilicity and the tensile strength can be tuned by appropriate selection of the polymers and the imparting of antioxidant and antimicrobial activity—by the incorporation of suitable low-molecular-weight bioactive agents. Notably, electrospinning is an effective technique for one-step and facile incorporation of a wide range of bioactive compounds (whether natural or synthetic) into polymer fibers under mild conditions, achieving high loading efficiency [3,4,5,6,7,8]. It has been claimed that the electrospun non-woven textile is an ideal material that meets the requirements for adequate wound healing, namely high porosity and surface area, good exudate absorption, adequate oxygen permeability, and prevention of bacterial penetration.

The dual spinneret electrospinning, in which two separate solutions of different compositions are electrospun simultaneously, represents a promising technique for the design of multifunctional fibrous materials. This approach results in composite materials with a complex architecture consisting of two types of fibers that differ in their composition and functionality. Herein, dual spinneret electrospinning was applied for the preparation of novel multifunctional fibrous materials composed of fibers from poly(L-lactide) (PLA)/lidocaine hydrochloride (LHC) and from poly(vinyl alcohol) (PVA)/chitosan (Ch)/rosmarinic acid (RA) (PLA/LHC + PVA/Ch/RA). To our knowledge, no data are available for composite materials built of these components. It was hypothesized that the prepared material would exhibit a combination of valuable properties, including adequate mechanical behavior and hydrophilicity, as well as antioxidant and antimicrobial activity due to the incorporated polyphenolic acid RA, and local anesthetic properties because of LHC. Having in mind the existing diverse techniques for the preparation of RA- and LHC-containing delivery systems, such as solid lipid nanoparticles, liposomes, and micro- and nanoemulsions [9,10,11,12,13], the electrospinning approach provides an alternative strategy that can overcome major drawbacks of these carriers, including limited drug loading capacity, low storage stability, high production costs, and potential toxicity due to the presence of surfactants.

It is expected that the composite non-woven textile will be composed of both types of fibers, distributed throughout the bulk and on the surface of the material. RA was selected because of the great variety of its bioactivities: antioxidant, antimicrobial, anti-inflammatory, wound healing, and anticancer properties [14,15,16]. Thus, RA is considered a promising antimicrobial and health-promoting agent that can find application in biomedicine as well as for extending the shelf life of food or in medical skincare products. There are data on the preparation of RA-loaded fibrous materials by blend electrospinning [17,18,19,20,21,22,23] or coaxial electrospinning [24]. To date, RA has not been incorporated in PVA/Ch fibers by blend electrospinning, nor in multifunctional materials by simultaneous dual electrospinning.

In the present study, Ch and PVA were selected as polymer partners of RA to prepare PVA/Ch/RA fibers by electrospinning due to their non-toxicity, biocompatibility, and solubility in aqueous medium [25,26,27]. Ch and PVA are biodegradable as well. Additionally, Ch has a valuable set of biological properties—antibacterial, hemostatic, antifungal, and anticancerogenic—as well as the ability to modulate the macrophage activity, thereby promoting accelerated wound healing [28,29,30,31]. A limitation of using PVA/Ch/RA fibers alone is their tendency to swell and dissolve in the exudate of injured tissue, resulting in disrupting the structural integrity of the non-woven textile. This is why it is necessary to obtain a multifunctional fibrous material in which, in addition to PVA/Ch/RA fibers, there are also fibers from the water-insoluble PLA. The latter would enable the preservation of the structural integrity of the prepared textile. PLA was selected for the following reasons: it is obtained from annually renewable sources; it is biocompatible, biodegradable, water-insoluble, and easily processed into fibers by electrospinning [32,33,34]. The presence of LHC (a local anesthetic [35,36]) in PLA fibers of the composite non-woven textile would confer pain-relieving properties, which are highly relevant for potential wound healing applications.

The present study aimed to prepare novel multifunctional composite fibrous materials composed of PLA or PLA/LHC fibers and PVA/Ch/RA fibers by simultaneous dual spinneret electrospinning. Due to the lack of data on the preparation of PVA/Ch/RA fibers by one-pot blend electrospinning, this type of fibrous material was obtained for the first time as well. It was expected that the novel composite materials would possess a set of valuable properties in terms of their applicability in wound healing: good structural resistance (due to the presence of PLA-based fibers); good draining of the wound (due to the fibers based on the PVA/Ch pair); antioxidant, antibacterial, and antifungal properties due to the incorporated bioactive components (Ch, RA, and LHC); and pain-relieving properties imparted by the incorporated LHC. The morphology of the novel materials was analyzed by scanning (SEM) and transmission (TEM) electron microscopy, as well as by confocal laser scanning microscopy (CLSM). In addition, the wettability, crystallinity, and thermal characteristics, as well as the mechanical properties of the prepared mats, were also investigated. The antioxidant activity of the obtained materials was evaluated by the DPPH^•^ test. The antimicrobial activity of the RA-loaded fibrous mats was tested in vitro against the pathogenic bacteria *Staphylococcus aureus* (*S. aureus*) and *Escherichia coli* (*E. coli*) and the fungi *Candida albicans* (*C. albicans*).

## 2. Materials and Methods

### 2.1. Materials

Poly(vinyl alcohol) (PVA) (M_w_ approx. 85,000–124,000 g·mol^−1^, 96% hydrolyzed, Acros Organics, Geel, Belgium), poly(L-lactide) (PLA) (M_n_ = 55,000 g·mol^−1^, M_w_/M_n_ = 1.75, Ingeo™ Biopolymer 6201D, NatureWorks, Minnetonka, MN, USA), and chitosan (Ch) (average viscometric molar mass 380,000 g·mol^−1^, deacetylation degree 80%, Aldrich, St. Louis, MO, USA) were used. Lidocaine hydrochloride (LHC) (Aldrich, St. Louis, MO, USA), rosmarinic acid (RA) (Merck, Billerica, MA, USA), 2,2-diphenyl-1-picrylhydrazyl free radical (DPPH^•^) (Sigma-Aldrich, Darmstadt, Germany), and fluorescein (free acid) (Merck, Billerica, MA, USA) were used as received. Dichloromethane (DCM) (Fisher Scientific UK, Loughborough, UK), dimethyl sulfoxide (DMSO) (Fisher Scientific UK, Loughborough, UK), glacial acetic acid (Merck, Billerica, MA, USA), and absolute ethanol (Merck, Billerica, MA, USA) were of analytical grade purity. Salts used for the preparation of the buffer solution of pH 7.4 (KH_2_PO_4_, Na_2_HPO_4_) were purchased from Merck Chemicals (Merck, Billerica, MA, USA).

*Staphylococcus aureus* (*S. aureus*) 3359, *Escherichia coli* (*E. coli*) 3397, and *Candida albicans* (*C. albicans*) 74 were supplied by the National Bank for Industrial Microorganisms and Cell Cultures (NBIMCC), Sofia, Bulgaria.

### 2.2. Preparation of the Electrospun Materials

#### 2.2.1. Preparation of PVA/Ch and RA-Containing PVA/Ch Mats by One-Pot Blend Electrospinning

For the preparation of PVA/Ch solution, Ch (2 wt%) was dissolved in 30% *v/v* acetic acid aqueous solution under continuous stirring for 8 h at room temperature. PVA solution with a concentration of 10 wt% was also obtained by dissolving the required amount of PVA powder in aqueous acetic acid (30% *v*/*v*) at 80 °C for 5 h. The solutions were then mixed to prepare a PVA/Ch solution at a weight ratio of PVA solution/Ch solution = 8:2. PVA mats prepared by electrospinning of PVA spinning solution (10 wt%) in aqueous acetic acid (30% *v*/*v*) were used as control ones.

To obtain PVA/Ch/RA mats, 0.252 g RA dissolved in 1.7 mL acetic acid aqueous solution (30% *v*/*v*) was added to 0.12 g Ch (3.0 wt%) solution in aqueous acetic acid (30% *v*/*v*). The solution was stirred for 5 h. For the preparation of PVA/Ch/RA solution with RA content 10 wt% (relative to the polymer weight) and PVA solution/Ch solution = 8:2 *w*/*w*, 24 g of 10 wt% PVA solution in aqueous acetic acid (30% *v*/*v*) was added to the prepared Ch/RA solution and agitated for 5 h. PVA/Ch mats containing different amounts of RA (5 and 7 wt% relative to the polymer weight) were also obtained using the same procedure. The electrospinning was performed under the following conditions: constant feed rate of 0.5 mL/h obtained by using an infusion pump (NE-300 Just InfusionTM Syringe Pump, New Era Pump Systems Inc., Farmingdale, NY, USA), applied voltage of 20 kV (high-voltage power supply for scientific use, Model: HVG-CONT-LCD, Linari Engineering [Pisa, Italy]), deposition time of 28 h, tip-to-collector distance of 15 cm, use of a grounded rotating aluminum drum collector with rotation speed maintained at 1300 rpm, room temperature of 21 °C, and a relative humidity of 49%. The obtained fibrous materials were dried under reduced pressure at 45 °C for 24 h.

#### 2.2.2. Preparation of Composite Fibrous Materials by Dual Spinneret Electrospinning

For comparison, PLA and PLA/LHC mats were prepared by one-pot electrospinning. PLA mats were obtained by electrospinning of PLA solution in a mixed DCM/DMSO solvent [75/25 (*w*/*w*), PLA 10 wt%]. To prepare LHC-containing PLA mats, electrospinning of a blend solution of PLA and LHC (LHC concentration was 10% relative to the polymer weight) in DCM/DMSO [75/25 (*w*/*w*)] at a total polymer concentration of 10 wt% was performed.

Two types of composite fibrous materials were prepared by applying the dual spinneret electrospinning technique. The first one (designated as (PLA + PVA/Ch/RA)) consisted of PLA fibers and PVA/Ch/RA fibers. The second type of material (denoted as (PLA/LHC + PVA/Ch/RA)) was composed of PLA/LHC fibers and PVA/Ch/RA fibers. To prepare these materials, two infusion pumps for delivering the solutions were used. For obtaining (PLA + PVA/Ch/RA) mats and (PLA/LHC + PVA/Ch/RA) mats, two separate solutions were prepared: (i) PLA solution—10 wt% in DCM/DMSO [75/25 (*w*/*w*)] and (ii) PVA/Ch/RA (10 wt% relative to the polymer weight) solution—the preparation is described in Section 2.2.1. or (iii) PLA/LHC solution (10 wt% PLA and 10 wt% LHC in DCM/DMSO [75/25 (*w*/*w*)] and (iv) PVA/Ch/RA (10 wt% RA) solution—the preparation is described in Section 2.2.1. The concentration of the components for the preparation of the solutions subjected to simultaneous dual spinneret electrospinning for the fabrication of the multifunctional composite fibrous materials is presented in Table S1, Supplementary Materials. For confocal laser scanning microscope observations two composite fibrous materials—(PLA/fluorescein (0.5 wt% fluorescein) + PVA/Ch/RA (10 wt% RA)) and (PLA/LHC/fluorescein (0.5 wt% fluorescein) + PVA/Ch/RA (10 wt% RA))—were fabricated. All solutions were prepared by the above-described procedures. A schematic illustration of the dual spinneret electrospinning setup used for the preparation of multifunctional composite materials is shown in Scheme S1 (Supplementary Materials). The two pumps (NE-300 Just Infusion^TM^ Syringe Pump, New Era Pump Systems Inc., Farmingdale, NY, USA) for delivering the solutions were positioned on both sides of the rotating collector (1300 rpm) at a 180° angle. The solution of PLA (PLA/fluorescein) or of PLA/LHC (PLA/LHC/fluorescein) was delivered at a rate of 1 mL/h, whereas the PVA/Ch/RA solution was delivered at a rate of 0.5 mL/h. Electrospinnings of PLA (PLA/fluorescein) and PLA/LHC (PLA/LHC/fluorescein) solutions were carried out at a voltage of 25 kV (high-voltage power supply for scientific use, Model: HVG-CONT-LCD, Linari Engineering [Pisa, Italy]), whereas PVA/Ch/RA solution was carried out at a voltage of 20 kV (high-voltage power supply for scientific use, Model: HVG-CONT-LCD, Linari Engineering [Pisa, Italy]. The distance between the nozzle tip and the rotating drum was 17 cm for PLA (PLA/fluorescein) and PLA/LHC (PLA/LHC/fluorescein) solutions and 15 cm for the PVA/Ch/RA solution. (PLA + PVA/Ch/RA) and (PLA/LHC + PVA/Ch/RA) mats were electrospun for 10 h. (PLA/fluorescein + PVA/Ch/RA) and (PLA/LHC/fluorescein + PVA/Ch/RA) mats were collected on a rotating collector over a duration of 1.0 h. The prepared fibrous materials were dried in a heated vacuum desiccator (Vacuo-Temp, J.P. Selecta, Abrera, Barcelona, Spain) at 45 °C for 24 h.

### 2.3. Characterization

A Brookfield DV-II+ Pro programmable viscometer (Middleboro, MA, USA) for the cone/plate option, which has a cone spindle and a sample thermostated cup, was used to determine the dynamic viscosity of the spinning solutions at 25 ± 0.1 °C. The conductivity of the spinning solutions was measured in an electrolytic cell fitted with two rectangular platinum electrodes having a surface area of 0.6 cm^2^ as previously described [37]. Cell calibration was carried out using KCl standard solutions.

Scanning electron microscopy (SEM) was used for evaluating the morphology of the fabricated fibrous materials. Prior to SEM observation by a Jeol JSM-5510 SEM (Tokyo, Japan), the fibrous samples were coated with gold under high vacuum. The mean fiber diameter and the standard deviation were determined by applying ImageJ software (V.1.53 e, Wayne Rasband, National Institute of Health, Bethesda, MD, USA). For this reason, at least 40 counts of fibers from two different SEM images of each fibrous sample were performed. The JEM 2100 (JEOL Ltd., Tokyo, Japan) operating at 200 kV was applied to perform TEM analyses.

The Leica TCS SPE confocal laser scanning microscope (CLSM, Wetzlar, Germany) was used to observe the fibrous structure of the obtained (PLA/fluorescein (0.5 wt% fluorescein) + PVA/Ch/RA (10 wt% RA)), (PLA/LHC/fluorescein (0.5 wt% fluorescein) + PVA/Ch/RA (10 wt% RA)), PVA/Ch/RA (10 wt% RA), and PLA/LHC/fluorescein (0.5 wt% fluorescein) electrospun materials. This microscope was equipped with a Leica Application Suite X (LAS X) Microscope Software, Version 3.5.7.23225 (Leica Microsystems CMS GmbH, Wetzlar, Germany). The fibrous samples were cut to 10 × 10 mm^2^ and put onto a glass slide. Two drops of immersion oil (Sigma-Aldrich, St. Louis, MO, USA) were placed on the sample surface and left until complete wetting occurred. The wetted samples were then mounted with a cover glass. The immersion oil has a refractive index of 1.516, and its role is to achieve high magnification imaging. All images were prepared with an objective ACS APO 63× (oil immersion; a drop of immersion oil was placed between the cover glass and the objective) and a digital zoom factor of 3×. The RA- and fluorescein-loaded mats were excited with 405 nm and 488 nm lasers, respectively. In this case, emissions at 430–460 nm and 530–560 nm were used in the first and second channels, respectively. Two detection channels were employed to collect fluorescence from two lasers successively for each Z-layer. The fluorescence emission from RA- and fluorescein-loaded mats was registered by a Leica DM5500Q confocal spectral detector TCS SPE L (Wetzlar, Germany). The pixel resolution of the image was 2048 × 2048; the image size was 58.2 × 58.2 μm^2^, and the digital resolution is 0.03 μm/pixel. The scanning was carried out from the top layer of each electrospun fibrous sample towards a depth of 15 µm with a Z-step of 1 µm, and the total laser exposure was 8.4 min.

The static sessile drop method was applied to determine the water contact angles (WCA) of the fibrous materials. These WCA measurements were carried out by using an Easy Drop DSA20E Krüss GmbH apparatus (Hamburg, Germany). A sessile droplet of distilled water (10 μL) was placed on the fibrous mat surface. WCA mean values were ascertained by computer analysis of the droplet’s temporal images based on at least 20 measurements.

The assessment of the amorphous/crystalline structure of fabricated mats was conducted employing X-ray diffraction (XRD) analyses at 2*θ* range from 5° to 60° with a step of 0.02°. The counting time was 1 s/step. These analyses were performed on a computer-controlled D8 Bruker Advance powder diffractometer (Billerica, MA, USA) with a LynxEye detector and employing filtered Cu Kα radiation (λ = 1.5406 Å) at room temperature.

Attenuated total reflection-Fourier transform infrared (ATR-FTIR) measurements were conducted on a Nicolet™ iS™50 spectrometer (Thermo Fisher Scientific Inc., Waltham, MA, USA) at a spectral range of 4000–400 cm^−1^ using OMNIC software, version 1.04. This spectrometer was fitted with an IR diamond iS50 ATR (Thermo Fisher Scientific Inc., Waltham, MA, USA) (diamond crystal) accessory.

In order to determine the thermal behavior of the obtained materials, differential scanning calorimetry (DSC) analyses using Discovery DSC 250 (TA, Instruments, New Castle, DE, USA) were carried out. Measurements were conducted in the temperature range from 20 to 200 °C using a nitrogen flow and a heating rate of 10 °C/min. Melting temperatures (T_m_) and the fusion enthalpies (ΔH_m_) were calculated using the first heating run. The degree of crystallinity of PLA (χ_c_^PLA^, %) and PVA (χ_c_^PVA^, %) in the fibrous materials was determined using Equations (1) and (2):

(1) $$\chi_{c}^{\mathrm{PLA}},\%=\left[\frac{\left(\Delta H_{m}^{\mathrm{PLA}}-\Delta H_{cc}^{\mathrm{PLA}}\right)}{\left(\Delta H_{m}^{\mathrm{PLA},0}\times {\;W}^{\mathrm{PLA}}\right)}\right]\times 100,$$

(2) $$\chi_{c}^{\mathrm{PVA}},\%=\left[\frac{{\Delta H}_{m}^{\mathrm{PVA}}}{\left({\Delta H}_{m}^{\mathrm{PVA},0}\times {\;W}^{\mathrm{PVA}}\right)}\right]\times 100,$$

where W^PLA^(W^PVA^) is the mass fraction of PLA (PVA) in the mats; ΔH_m_^PLA^ (ΔH_m_^PVA^) is the PLA (PVA) enthalpy of fusion in the mats; ΔH_cc_^PLA^ is the enthalpy corresponding to the temperature of cold crystallization; ΔH^0^_m_ is the enthalpy of fusion, when the respective polymer is in a 100% crystalline state; ΔH_m_^PLA,0^ = 93.0 J/g [38]; and ΔH_m_^PVA,0^ = 138.6 J/g [39].

X-ray photoelectron spectroscopy (XPS) was performed on an ESCALAB-MkII (VG Scientific, East Grinstead, UK) electron spectrometer fitted with an ultrahigh-vacuum (UHV) chamber. The spectra were obtained using an Mg Kα X-ray source. All high-resolution spectra were energy calibrated using the C_1s_ line at 285 eV as a reference. The data were processed by using CasaXPS software (version 2.3.25PR1-0). Peak fitting was performed with Gauss–Lorenzian-type line shapes after subtraction of the Shirley background.

Tensile tests were conducted at room temperature to determine the mechanical properties of PVA/Ch/RA, (PLA + PVA/Ch/RA), and (PLA/LHC + PVA/Ch/RA) fibrous materials using an INSTRON 3344 apparatus (load cell of 50 N, stretching rate of 20 mm/min). Mat specimens (20 mm × 60 mm (width × length)) were cut in such a way that their length was along the collector rotation direction. The thickness of the fibrous samples was measured by a Digital Thickness Gauge FD 50 (Käfer GmbH, Bremen, Germany). The obtained specimen was fixed with two clamps with an initial length between them of 40 mm. Ten specimens of each fibrous material were tested, and averages of Young’s modulus, tensile strength, and elongation at break were assessed.

The weight losses of PVA/Ch/RA, (PLA + PVA/Ch/RA), and (PLA/LHC + PVA/Ch/RA) mats in PBS of pH 7.4 were estimated using specifically designed cells, described in our previous studies [40], for the fixation of the mats. The mats were immersed in PBS for 24 h and 48 h, then rinsed with water and freeze-dried. The morphology of the samples treated for 24 h and 48 h was analyzed using a Jeol JSM-5510 SEM microscope (Tokyo, Japan).

The swelling degree (α) of electrospun (PLA + PVA/Ch/RA) and (PLA/LHC + PVA/Ch/RA) composite mats in PBS was studied by measuring gravimetrically the change of the weight over time and was calculated using Equation (3):

α% = (weight of swollen mats − weight of dry mats)/weight of dry mats × 100(3)

### 2.4. In Vitro RA and/or LHC Release

The in vitro RA and/or LHC release was conducted in the following way: 25 mg PVA/Ch/RA mat, 75 mg (PLA + PVA/Ch/RA) mat, 75 mg (PLA/LHC + PVA/Ch/RA) mat, and 50 mg PLA/LHC mat were immersed in 100 mL of PBS (pH 7.4, ionic strength of 0.1) at 37 °C and stirred at 100 rpm. Aliquots of 2 mL were drawn out at specific time intervals and replaced with the same amount of PBS. The absorbance of aliquots was measured by a DU 800 UV-Vis spectrophotometer produced by Beckman Coulter, Brea, CA, USA, at a wavelength of 324 nm for RA-loaded mats and of 261 nm for LHC-loaded mats, respectively. The calibration curves (correlation coefficient R~0.999) for RA or LHC in PBS were used to determine the amount of the released RA or LHC over time. The data are mean values of three measurements. The total amount of RA or LHC in the fibrous materials was determined by a DU 800 UV spectrophotometer (Beckman Coulter, Brea, CA, USA), after immersing three fibrous samples of known weight in 20 mL of ethanol for 6 h under stirring. The amount of drug was calculated by UV measurements at a wavelength of 327 nm and 262 nm for RA and LHC, respectively. The amount of the incorporated drugs was found to be equal to that present in the spinning solution.

The release kinetics of RA and LHC were evaluated using the Korsmeyer–Peppas model [41]:

M_t_/M_∞_ = Kt^n^ (4)

where M_t_ is the amount of drug released at time t, M_∞_ is the total amount of drug loaded in the fibrous materials, K is the release rate constant, and n is the release exponent.

### 2.5. Estimation of the Antioxidant Capacity of the Fibrous Materials

The antioxidant capacity of the electrospun fibrous materials containing RA and/or LHC was evaluated using the DPPH^•^ assay. For this assay, a DPPH^•^ solution in ethanol (0.1 × 10^−3^ M) was prepared. 0.5 mL of RA solution in ethanol with a concentration of 5 × 10^−3^ M or 0.5 mL of LHC solution in ethanol (concentration of 1.33 × 10^−2^ M) was added to 3 mL of DPPH^•^ solution in ethanol. 0.0066 g of PVA/Ch/RA mats, 0.0198 g of (PLA + PVA/Ch/RA) or (PLA/LHC + PVA/Ch/RA) mats, or 0.0132 g of PLA/LHC mats were immersed in 2 mL of DPPH^•^ solution in ethanol. Each solution stays in the dark for half an hour. Then, the absorbance of the DPPH^•^ radical solution was measured at 517 nm by a DU 800 UV-Vis spectrophotometer (Beckman Coulter, Brea, CA, USA). The procedure was performed three times. The antioxidant activity (AA, %) was determined as follows:

(5) $$\mathrm{Inhibition},\mathrm{AA},\%=\left[\frac{\left(A_{{\mathrm{DPPH}}^{•}}-A_{\mathrm{sample}}\right)}{A_{{\mathrm{DPPH}}^{•}}}\right]\times 100$$

where A_DPPH^•^_ is the absorbance of DPPH^•^ at 517 nm and A_sample_ is the absorbance of DPPH^•^ solution mixed with sample solution at 517 nm.

### 2.6. Assessment of the Antibacterial and Antifungal Activity of the Fibrous Materials

The antibacterial and antifungal activity of the prepared fibrous materials against the bacteria *S. aureus* 3359 and *E. coli* 3397 and the fungi *C. albicans* 74 was investigated in vitro in liquid medium by using the viable cell-counting method. *S. aureus* 3359 and *E. coli* 3397 were pre-cultured on Tryptic Soy Agar medium (TSA, Becton Dickinson, Heidelberg, Germany) and incubated at 37 °C for 24 h. *C. albicans* 74 was pre-cultured on Sabouraud Dextrose Agar (SDA, Becton Dickinson, Sparks, MD, USA) medium and incubated at 37 °C for 48 h. Prior to the experiment, all tested mats were sterilized using UV light. A weighted amount of fibrous samples was immersed in bacterial (fungal) suspension with a concentration of ca. 10^5^ cells/mL, prepared in tubes with 1 mL of 0.9% saline solution. Then these tubes were incubated at 37 °C. At defined durations of time (3, 5, and 24 h), 50 μL of sample was taken out of each tube and serially diluted ten times using sterile 0.9% saline solution. After dilutions, the aliquots of 50 μL were plated on Petri dishes with TSA (Becton Dickinson, Heidelberg, Germany) solid medium for *S. aureus* and *E. coli* and SDA (Becton Dickinson, Sparks, MD, USA) solid medium for *C. albicans*. The plates were incubated for 24 h at 37 °C for the test with bacteria and for 48 h at 37 °C for the test with fungi *C. albicans*. The number of the surviving bacteria or fungi was calculated by counting the colony-forming units (CFU). Counting was carried out three times for each experiment.

### 2.7. SEM Observation of the Adhesion of S. aureus Cells to the Surface of Fibrous Materials

SEM observation of pathogenic bacterial *S. aureus* cells adhered to the surface of mats from PLA and those from (PLA + PVA/Ch/RA) and (PLA/LHC + PVA/Ch/RA) after incubation with a bacteria suspension was conducted in order to investigate the interaction of tested mats with *S. aureus.* The fibrous materials were incubated at 37 °C in 2 mL of the *S. aureus* culture, which contained 6.8 × 10^5^ cells/mL, for 24 h. Saline solution (0.9% NaCl) was then used to wash the samples twice in order to remove any bacteria that had not adhered. The bacteria that were adhered to the surface of the fibrous materials were fixed by immersing the mats in 2.5 wt% glutaraldehyde solution in saline solution at 4 °C for 5 h. The mats were then carefully washed with saline solution once more before being freeze-dried. The studied mats were coated with gold, and the morphology of the adhered bacteria *S. aureus* was detected by SEM Jeol JSM-5510 (Jeol Ltd., Tokyo, Japan).

### 2.8. Statistical Data Analysis

The results of the current study are displayed as means ± standard deviation (SD), and one-way analysis of variance (ANOVA) was utilized to assess the statistical significance of the data using GraphPad PRISM (Version 5, GraphPad Software Inc., San Diego, CA, USA). The following thresholds were employed to determine statistical significance: *p* < 0.05 (*), *p* < 0.01 (**), and *p* < 0.001 (***).

## 3. Results and Discussion

In the context of the design of novel multifunctional fibrous materials with potential biomedical applications, it was of particular interest to combine the beneficial properties of RA and/or LHC with those of Ch, PVA, and PLA using the electrospinning technique. The following approaches were applied: (i) electrospinning of a blend solution of PVA, Ch, and RA (for brevity, the obtained mats are denoted as PVA/Ch/RA); (ii) simultaneous dual spinneret electrospinning of a solution of PLA and a solution of PVA, Ch, and RA [designated as (PLA + PVA/Ch/RA) mats]; and (iii) simultaneous dual spinneret electrospinning of a solution of PLA and LHC and a solution of PVA, Ch, and RA [denoted as (PLA/LHC + PVA/Ch/RA) mats]. Unlike the fibrous materials obtained by electrospinning of a blend solution consisting of only one type of fiber, the composite materials obtained by dual spinneret electrospinning consist of two types of fibers—PVA/Ch/RA fibers and PLA or PLA/LHC fibers. For comparison, PVA, PVA/Ch, PLA, and PLA/LHC mats were also prepared by electrospinning.

### 3.1. Preparation of PVA/Ch/RA Fibrous Materials by One-Pot Blend Electrospinning and Characterization of Their Morphology

**Figure 1:**
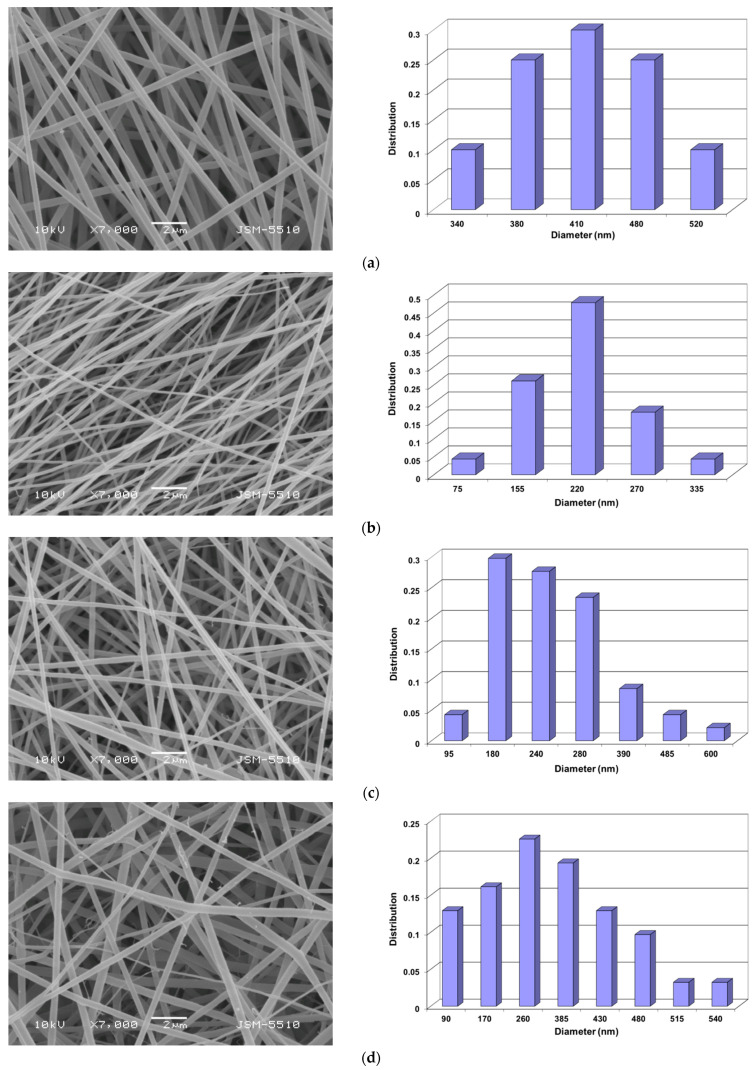

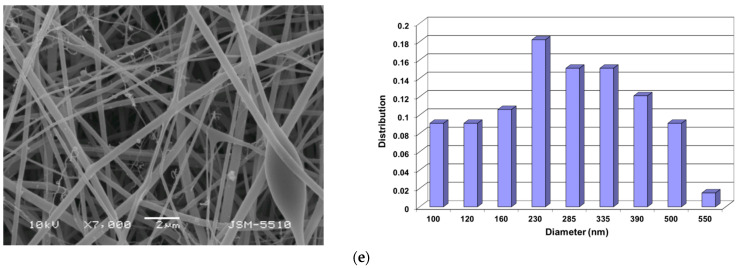
SEM micrographs and fiber diameter distributions of the fabricated electrospun mats: (**a**) PVA, (**b**) PVA/Ch, (**c**) PVA/Ch/RA (5 wt% RA), (**d**) PVA/Ch/RA (7 wt% RA), and (**e**) PVA/Ch/RA (10 wt% RA). SEM magnification: ×7000.

It is known that PVA is a suitable non-ionogenic partner, which can enable the preparation of Ch-containing fibers by electrospinning when added to a Ch solution [37,42,43,44,45]. Therefore, in the present study, PVA was selected to prepare the spinning solutions containing Ch. In previous studies, the effect of the PVA/Ch ratio on the morphology of the fibers obtained by electrospinning has been studied [46], and we have found that defect-free and continuous cylindrical fibers were obtained by electrospinning of a blend solution of the components at PVA solution/Ch solution = 8:2 (*w*/*w*). Therefore, solutions of PVA/Ch at PVA solution/Ch solution = 8:2 (*w*/*w*) were selected to prepare RA-containing PVA/Ch fibers. Furthermore, a suitable solvent system of 30% (*v*/*v*) aqueous acetic acid was used in order to enable the dissolution of Ch and RA as well as the successful electrospinning of PVA/Ch/RA blend solutions. As seen from the SEM micrographs presented in Figure 1, the electrospinning of PVA/Ch/RA solutions in 30% (*v*/*v*) aqueous acetic acid resulted in the preparation of continuous fibers that were cylindrical in shape. Defect-free fibers were obtained by electrospinning of PVA/Ch/RA solutions containing 5 or 7 wt% RA. A small number of spindle-like defects with average sizes of 2150 × 8100 nm were formed when 10 wt% RA was added to the spinning solutions. The determined RA loading efficiency in the mats containing 10 wt% RA was 97.8 ± 1.5%, which is very close to the theoretical one. Under the same electrospinning conditions, defect-free and cylindrical fibers of PVA and PVA/Ch were obtained. The mean diameter of the fibers prepared from PVA solution was 425 ± 60 nm (Figure 1a). PVA/Ch fibers (weight ratio PVA/Ch = 8:2) had a mean diameter of 210 ± 60 nm (Figure 1b). Therefore, the mean fiber diameter decreased with the addition of Ch to the PVA solution. This was most likely due to a decrease in the viscosity of the PVA solution in the presence of Ch (from 1530 cP to 1220 cP, Table S2). An increase in the conductivity of the PVA/Ch solution was also observed, which may have also contributed to the decrease in the mean fiber diameter (Supplementary Materials, Table S2). The incorporation of RA in PVA/Ch solutions resulted in a slight increase in the mean diameters of the fibrous mats (Figure 1c–e, Supplementary Materials, Table S2), which was most probably due to an increase in the viscosity of the PVA/Ch solutions containing RA as compared to the PVA/Ch spinning solution. The conductivity of the PVA/Ch solutions changed insignificantly with the addition of 5, 7, or 10 wt% RA (Supplementary Materials, Table S2). The observed increase in viscosity might be attributed to intermolecular interactions based on hydrogen bonds between PVA, Ch, and RA.

**Figure 2:**
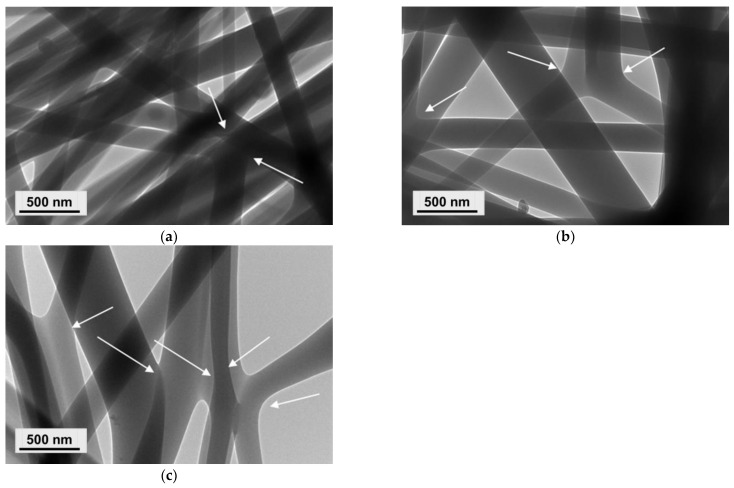
TEM micrographs of fibers from (**a**) PVA/Ch mat, (**b**) PVA/Ch/RA mat (5 wt% RA), and (**c**) PVA/Ch/RA mat (10 wt% RA). Magnification: ×10,000. The branching of some of the main fibers is indicated with an arrow.

In addition, PVA/Ch/RA fibers had a broader diameter distribution as compared to that of PVA and PVA/Ch fibers. As the amount of incorporated RA in the PVA/Ch fibers increased, a further broadening in the fiber diameter distribution was detected. This also explains the observed increase in the standard deviation of the mean diameters of PVA/Ch/RA fibers (Supplementary Materials, Table S2). The TEM micrographs presented in Figure 2a revealed the presence of some fiber branching in the case of PVA/Ch fibers. Similar results have also been reported by other authors for electrospun Ch and Ch/PLA fibers using trifluoroacetic acid as a solvent [47,48]. The performed TEM analyses also showed that the increase in RA amount in the PVA/Ch/RA solutions had an additional effect on the fiber morphology (Figure 2b,c). In these systems, the process of fiber branching was more pronounced than in the PVA/Ch system. Moreover, the SEM micrographs (Figure 1c–e) clearly showed the appearance of very thin fiber branches originating from the main fibers in the PVA/Ch/RA fibrous materials. The observed branching of the fibers was most likely attributed to an ionic imbalance induced by both the ionogenic polymer Ch and the low-molecular-weight acid RA on the surface of the jet during the electrospinning process [49,50]. A similar effect has also been evidenced during the electrospinning of solutions of non-ionogenic polymers in the presence of a low-molecular-weight salt [51], as well as during the electrospinning of *N*-carboxyethylchitosan/poly(ethylene oxide)/AgNO_3_ systems [52] and poly(ethylene oxide)/PLA/beeswax/5-nitro-8-hydroxyquinoline systems [53].

The weight losses of PVA/Ch and PVA/Ch/RA fibrous materials were estimated using a specially designed cell, described in our previous study [40], after 24 and 48 h stay in PBS buffer. The experimentally determined weight losses of the PVA/Ch and PVA/Ch/RA (10 wt% RA) mats after 24 h were 78% and 85%, respectively. The weight losses after a 48 h stay in the buffer solution were 88.9% and 92% for the PVA/Ch and PVA/Ch/RA mats, respectively. These values were lower than the theoretically calculated ones of 95.24% and 95.67% for the PVA/Ch and PVA/Ch/RA mats, respectively, estimated based on the assumption that PVA and RA were completely dissolved after the 24 h and 48 h stay of the materials in the buffer solution. The obtained results showed that after the stay in the PBS buffer solution, a certain amount of PVA remained in the fibrous materials.

### 3.2. Preparation of Composite Fibrous Materials from PVA/Ch/RA Fibers and PLA or PLA/LHC Fibers by Dual Spinneret Electrospinning and Characterization of Their Morphology

**Figure 3:**
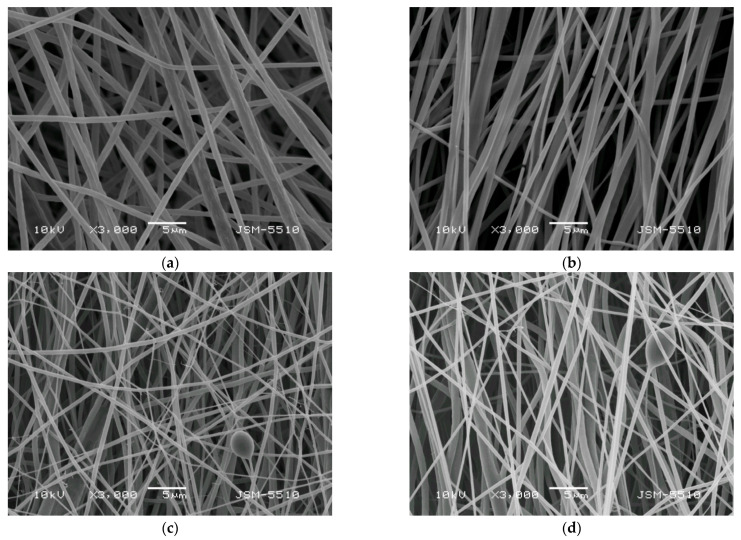
SEM micrographs of the fabricated electrospun mats: (**a**) PLA, (**b**) PLA/LHC, (**c**) (PLA + PVA/Ch/RA), and (**d**) (PLA/LHC + PVA/Ch/RA). SEM magnification: ×3000.

In order to prepare fibrous mats of different composition and design combining the valuable properties of Ch, PVA, and PLA and the favorable biological properties of RA and/or LHC, the simultaneous dual spinneret electrospinning technique of two solutions was used. Thus, (PLA + PVA/Ch/RA) and (PLA/LHC + PVA/Ch/RA) composite fibrous materials were successfully obtained. For comparison, PLA and PLA/LHC fibers were also prepared. As seen from the SEM micrographs presented in Figure 3a, the electrospinning of a PLA solution led to the formation of fibrous materials composed of randomly arranged cylindrical and defect-free fibers with a mean diameter of 780 ± 250 nm. Upon addition of LHC to the PLA solution, a decrease in the mean fiber diameter (710 ± 234 nm) was detected (Figure 3b). In addition, a certain alignment of the fibers mainly along the axis of rotation of the rotating collector was also observed. This might be attributed to the higher conductivity of PLA solutions containing LHC because of LHC’s ionic nature. It increased to 322 μS/cm for PLA/LHC solutions, while for the PLA solution, it was 6.4 μS/cm. In the (PLA + PVA/Ch/RA) composite mats obtained by simultaneous electrospinning of PLA and PVA/Ch/RA solutions, both the presence of thin fibers with a mean diameter of 330 ± 120 nm and a small number of spindle-like defects with an average size of 2800 × 4200 nm and fibers with larger mean diameters of 880 ± 240 nm were observed (Figure 3c). In the case of (PLA/LHC + PVA/Ch/RA) composite fibers, in addition to the thin fibers with a mean diameter of 290 ± 112 nm and a small number of spindle-like defects with an average size of 3500 × 6150 nm, thicker fibers with a mean diameter of 790 ± 220 nm were also detected (Figure 3d). In addition, in the composite fibrous materials, branching of the thin fibers was also detected (Figure 3c,d).

To date, very few studies have employed CLSM to investigate electrospun fibrous materials [54,55,56]. CLSM is a non-destructive optical imaging technique that, unlike SEM and TEM, allows the investigation of fibrous material morphology to a depth of several tens of μm along the z-axis. CLSM microscopy was applied to demonstrate the simultaneous presence of both types of fibers—PLA fibers and PVA/Ch/RA fibers in the (PLA + PVA/Ch/RA) composite mats, as well as PLA/LHC fibers and PVA/Ch/RA fibers in the (PLA/LHC + PVA/Ch/RA) composite mats, both on the surface and within the bulk of the composite fibrous materials, as well as to investigate the morphology of these fibers. Control PVA/Ch/RA and PLA/LHC mats were also examined by CLSM microscopy. The CLSM studies were carried out by scanning the individual Z-layers of the materials, starting from the top layer of the fibrous sample, with a Z-step of 1 μm, to a depth of 15 μm. As shown in the CLSM micrographs presented in Figure S1 (Supplementary Materials), the fibers of the control PVA/Ch/RA mat, excited by blue laser photons at a wavelength of 405 nm, were observed by CLSM as colored in blue, which is due to the fluorescent emission of the RA incorporated into the fibers. RA possesses intrinsic fluorescence.

**Figure 4:**
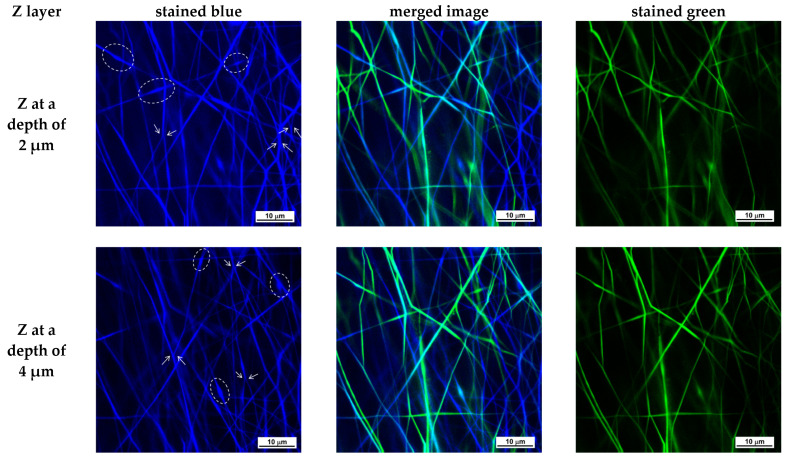

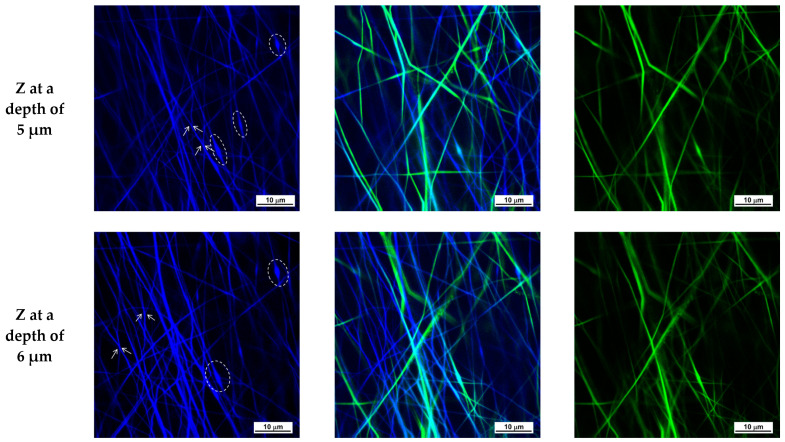
Confocal laser scanning microscopy (CLSM) images of composite fibers from [PLA/fluorescein (0.5 wt%)]/[PVA/Ch/RA (10 wt%)]. PVA/Ch/RA fibers are stained in blue by the embedded RA, and PLA/fluorescein fibers are stained in green by the embedded fluorescein. Selected images of Z-layers at a depth of 2 µm, 4 µm, 5 µm, and 6 µm are shown. The Z step of the images is 1 μm. Scale bars = 10 µm. The branching of some of the main fibers is indicated with arrows, and the spindle-like defects are marked with dotted lines.

**Figure 5:**
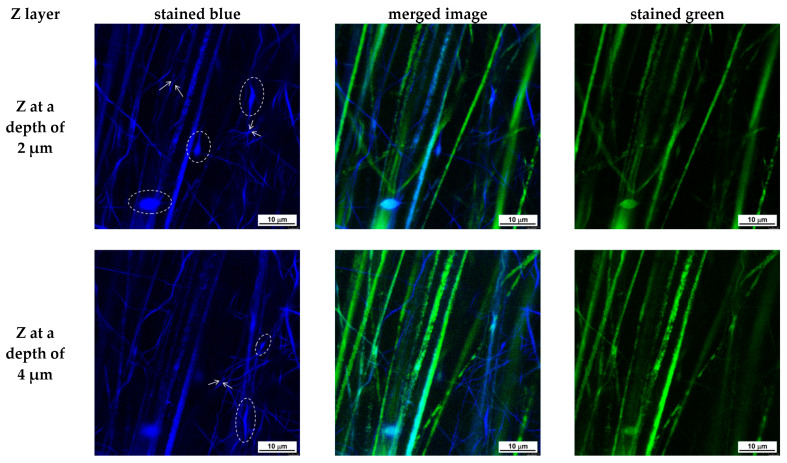

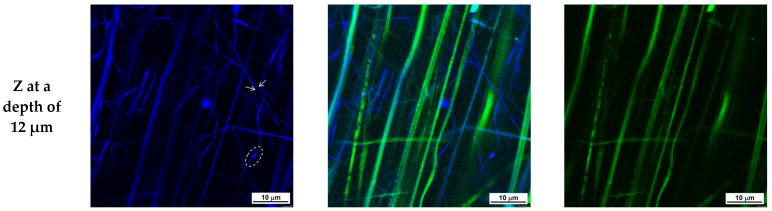
CLSM images of composite fibers from [PLA/LHC/fluorescein (0.5 wt%)]/[PVA/Ch/RA (10 wt%)]. PVA/Ch/RA fibers are stained in blue by the embedded RA, and PLA/LHC/fluorescein fibers—stained in green by the embedded fluorescein. Selected images of the Z-layer at a depth of 2 µm, 4 µm, and 12 µm. The Z step of the images is 1 μm. Scale bars = 10 µm. The branching of some of the main fibers is indicated with arrows, and the spindle-like defects are marked with dotted lines.

As seen in Figure S1 (Supplementary Materials), the fluorescence intensity is uniform along the length of the PVA/Ch/RA fibers within a given Z-layer. This indicates that the RA incorporated into the PVA/Ch/RA fibers, located either on the surface or within the bulk of the studied fibrous sample at a depth of 15 μm, is uniformly distributed within the fiber structure. As observed by SEM and TEM, the analysis by CLSM also revealed the branching of the main fibers, as well as a small number of spindle-like defects (Supplementary Materials, Figure S1). In the control PLA/LHC mat, no fluorescence was detected during observation by CLSM. Therefore, to visualize the fibers of this mat, it was necessary to incorporate a fluorescent dye into its structure. We selected fluorescein (free acid) as a fluorescent marker. It was incorporated into the spinning solution of PLA/LHC. The obtained solution was subjected to electrospinning. The green laser at a wavelength of 488 nm in CLSM excited the fluorescein incorporated into the PLA/LHC fibers, and the corresponding emission was observed (Supplementary Materials, Figure S2). The difference between the emissions of RA in the PVA/Ch/RA fibers and fluorescein in the PLA/LHC/fluorescein fibers enabled the two types of fibers present in the composite electrospun materials to be visualized separately in different channels. A mat composed of PLA/fluorescein fibers and PVA/Ch/RA fibers was also prepared. Figure 4 and Figure 5 present CLSM micrographs of (PLA/fluorescein + PVA/Ch/RA) and (PLA/LHC/fluorescein + PVA/Ch/RA) mats, respectively, recorded for selected Z-layers at a defined depth (Z-step of 1 μm). On the left, the micrographs of the blue channel are shown; on the right, those of the green channel; and in the middle, the merged image. As seen in Figure 4 and Figure 5, in the composite mats the blue-colored fibers are the RA-containing PVA/Ch fibers, while the PLA or PLA/LHC fibers labeled with fluorescein are colored green. From the images of the blue and green channels, as well as from the merged image, it can be seen that in the composite fibrous materials, both types of fibers—PLA (PLA/LHC) (stained in green) and PVA/Ch/RA (stained in blue)—are present in the individual Z-layers of the materials at a depth of 15 μm. Furthermore, the use of CLSM enables the identification of certain morphological features of the constituent fibers within the bulk of the composite fibrous materials. From the images of the blue channel shown in Figure 4 and Figure 5, it can be seen that for the PVA/Ch/RA fibers in the composite fibrous materials, a small number of spindle-like defects, as well as branching in some of the main fibers, were detected. Similar features were not observed in the morphology of the PLA (PLA/LHC) fibers in the composite fibrous materials (Figure 4 and Figure 5).

In terms of the potential biomedical application of the obtained composite fibrous materials, their stability was studied after contact with PBS buffer (pH 7.4) for 24 and 48 h, respectively. The experimentally determined weight losses of the (PLA + PVA/Ch/RA) and (PLA/LHC + PVA/Ch/RA) composite mats after a 24 h stay in the buffer were 5% and 21.7%, respectively. The weight losses after a 48 h stay in the PBS buffer were 7.6% and 24.8% for (PLA + PVA/Ch/RA) and (PLA/LHC + PVA/Ch/RA) mats, respectively. The theoretical weight loss values, calculated based on the assumption that PVA, RA, and/or LHC were completely dissolved after the stay of the materials in the buffer solution, were 31.86% and 37.92% for the (PLA + PVA/Ch/RA) and (PLA/LHC + PVA/Ch/RA) composite mats, respectively. Therefore, the weight losses of the composite mats after a 24 h and 48 h stay in PBS buffer were lower than the theoretical ones. This is an indication that for this period of time, a certain amount of PVA remained within the fibrous materials. In addition, the weight losses of (PLA + PVA/Ch/RA) composites were lower than those of (PLA/LHC + PVA/Ch/RA) ones. This might be attributed to the smaller equilibrium swelling degree determined in PBS buffer at 25 °C for the (PLA + PVA/Ch/RA) mats (282%) compared to that of the (PLA/LHC + PVA/Ch/RA) mats (445%). As seen from the SEM micrographs presented in Figure S3 (Supplementary Materials), the fibers in the respective composite materials swelled, but even after staying in PBS buffer for 24 and 48 h, the fibrous structure of the mats was preserved. Therefore, the (PLA + PVA/Ch/RA) and (PLA/LHC + PVA/Ch/RA) composite mats possess improved behavior as compared to those of PVA/Ch/RA fibrous materials when kept for 48 h in PBS buffer solution.

### 3.3. ATR-FTIR Analysis of the Fibrous Materials

**Figure 6:**
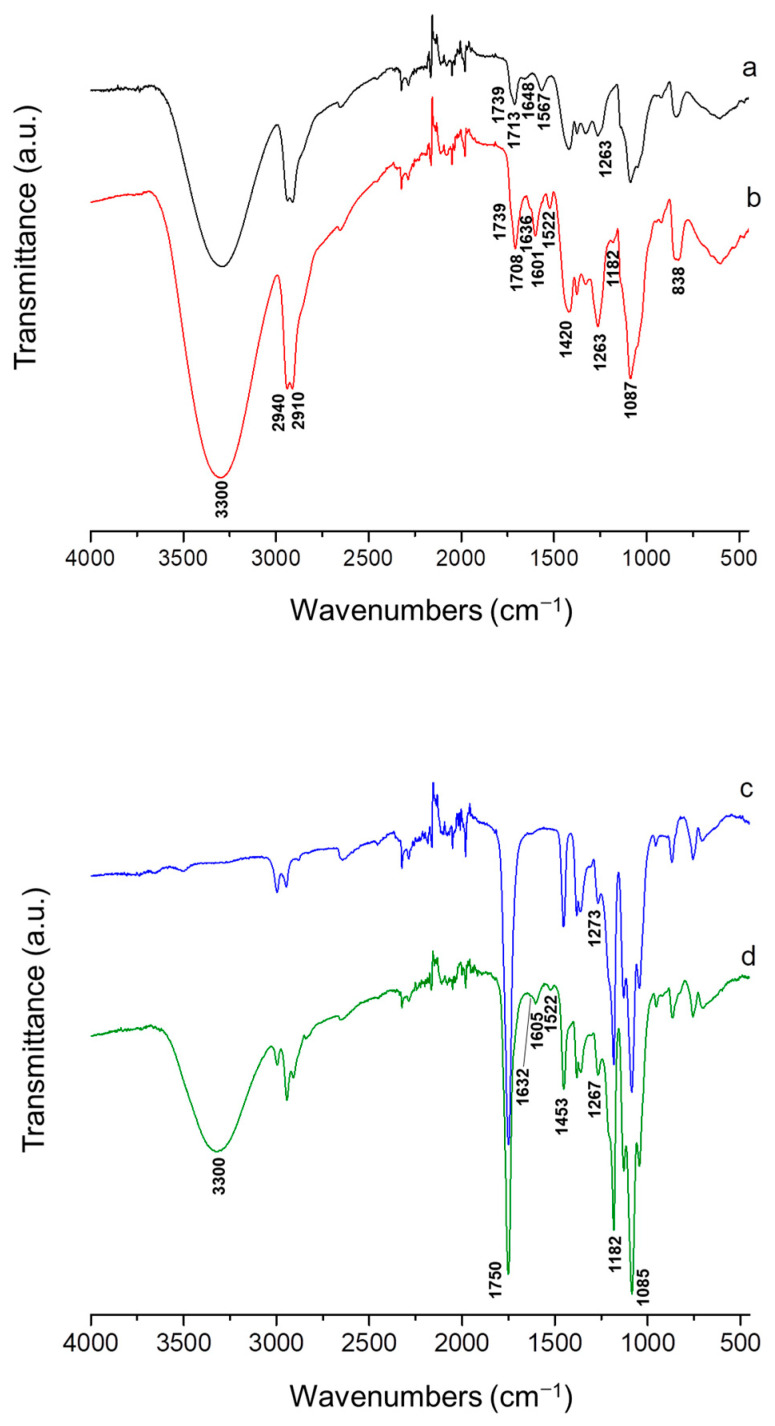

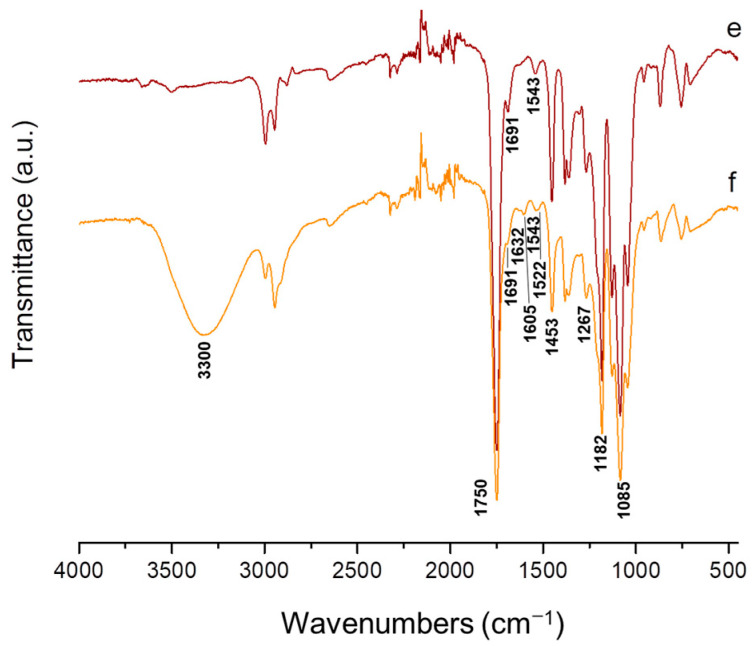
ATR–FTIR spectra of mats: (a) PVA/Ch, (b) PVA/Ch/RA (10 wt% RA), (c) PLA, (d) (PLA + PVA/Ch/RA), (e) PLA/LHC, and (f) (PLA/LHC + PVA/Ch/RA).

ATR-FTIR spectroscopy was conducted to characterize PVA/Ch and PVA/Ch/RA mats, as well as (PLA + PVA/Ch/RA) and (PLA/LHC + PVA/Ch/RA) composite mats. In the ATR-FTIR spectrum of PVA/Ch fibrous materials, characteristic bands for both polymers were detected—PVA (3300 cm^−1^—O-H stretching vibrations for PVA; 2940 and 2910 cm^−1^—aliphatic C-H stretching vibrations; 1739 cm^−1^ and 1713 cm^−1^—C=O stretching vibration from the residual vinyl acetate repeating units in PVA and from the residual acetyl groups in Ch; 1087 cm^−1^—C-O stretching vibration) [57] and Ch (1648 cm^−1^—amide I of the polysaccharide structure of Ch; 1567 cm^−1^—amide II; 3300 cm^−1^—O-H and N-H stretching vibrations) [57] (Figure 6a). In the spectrum of PVA/Ch/RA mats, in addition to the bands of PVA and Ch, new bands appeared: at 1708 cm^−1^, attributed to C=O stretching vibrations; at 1636 cm^−1^, characteristic of C=C stretching vibrations and C=O stretching vibrations; at 1601 cm^−1^, related to C=O stretching vibrations and C=C stretching vibrations, and at 1522 cm^−1^, characteristic of C-C stretching modes in the RA aromatic ring, accompanied by ring deformation [58] (Figure 6b). The bands in the spectrum of PVA/Ch/RA mats at 1601 cm^−1^ and 1636 cm^−1^ were shifted to lower wavenumbers by 6 cm^−1^ and by 11 cm^−1^, respectively, compared to those in the spectrum of RA at 1607 cm^−1^ and 1647 cm^−1^, respectively (Figure 6b and Supplementary Materials, Figure S4).

A shift of the band at 1522 cm^−1^ to a higher wavenumber by 6 cm^−1^ compared to that in the RA spectrum (1516 cm^−1^) was also detected (Figure 6b and Supplementary Materials, Figure S4). In addition, in the spectrum of PVA/Ch/RA mats, a band at 1182 cm^−1^ was recorded, characteristic of in-plane bendings of CH groups (δ_ip_C-H) (both aromatic and aliphatic ones) and in-plane bendings of OH groups (phenyl ones) from RA [58]. An increase in the intensity of the band at 1263 cm^−1^ due to δ_ip_C-H from RA was also detected. The band shifts registered in the ATR-FTIR spectra of PVA/Ch/RA mats indicate interactions between PVA, Ch, and RA, most likely arising from intermolecular hydrogen bonds between the partners.

PLA mats showed a strong band at 1750 cm^−1^ due to C=O stretching, a band at 1085 cm^−1^ characteristic of C-O-C stretching, and bands at 1453 cm^−1^ and 1382 cm^−1^ ascribed to –C-H bending of –CH(CH_3_)-groups (Figure 6c) [59]. In the spectrum of (PLA + PVA/Ch/RA) composite mats (Figure 6d), apart from the bands assigned to PLA, the appearance of new bands was recorded—a broad band at 3320 cm^−1^ due to O-H and N-H stretching of PVA and Ch, a band at 1632 cm^−1^ related to C=C stretching/C=O stretching of RA, a band at 1605 cm^−1^ characteristic of C=O stretching/C=C stretching of RA, a band at 1522 cm^−1^ for C-C stretching of the aromatic rings of RA, and a band at 1267 cm^−1^ assigned to in-plane bendings of C-H groups of RA [58].

As seen from Figure 6f, the spectrum of (PLA/LHC + PVA/Ch/RA) composite fibrous materials showed bands characteristic of PLA, PVA, Ch, and RA. In addition, bands assigned to LHC were also detected—a band at 1691 cm^−1^ ascribed to C=O stretching of the amide group and two sharp bands in the range 1450–1550 cm^−1^ related to C-N stretching [60]. The same bands characteristic of the drug LHC (Supplementary Materials, Figure S5) were also observed in the spectrum of LHC-containing PLA mats (Figure 6e). In the case of (PLA + PVA/Ch/RA) and (PLA/LHC + PVA/Ch/RA) composite mats, a number of bands were shifted, namely, the band at 1632 cm^−1^ (15 cm^−1^ to lower wavenumber), the band at 1605 cm^−1^ (2 cm^−1^ to lower wavenumber), and the band at 1522 cm^−1^ (6 cm^−1^ to higher wavenumber) compared to RA (Figure 6d,f and Supplementary Materials, Figure S4). Therefore, it can be assumed that interactions between PVA, Ch, and RA occurred in the obtained composite mats. The results from the performed ATR-FTIR analyses also confirmed the successful preparation of (PLA + PVA/Ch/RA) and (PLA/LHC + PVA/Ch/RA) composite materials by applying dual spinneret electrospinning.

### 3.4. Thermal Behavior of the Fibrous Materials

**Figure 7:**
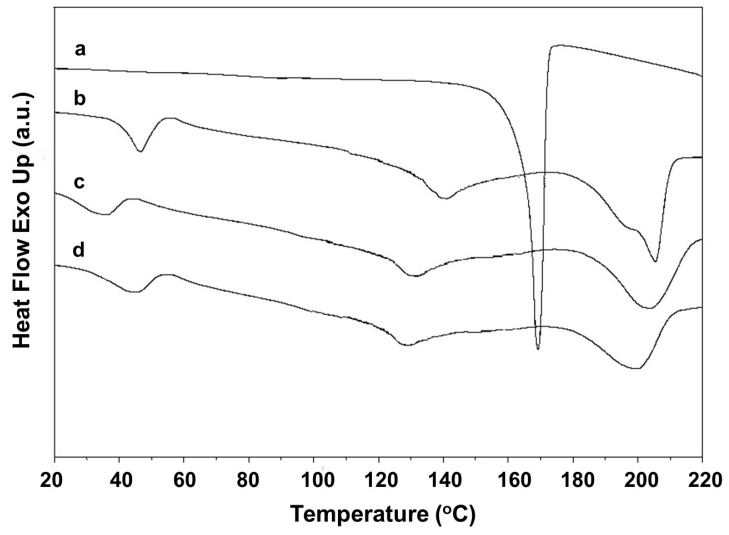

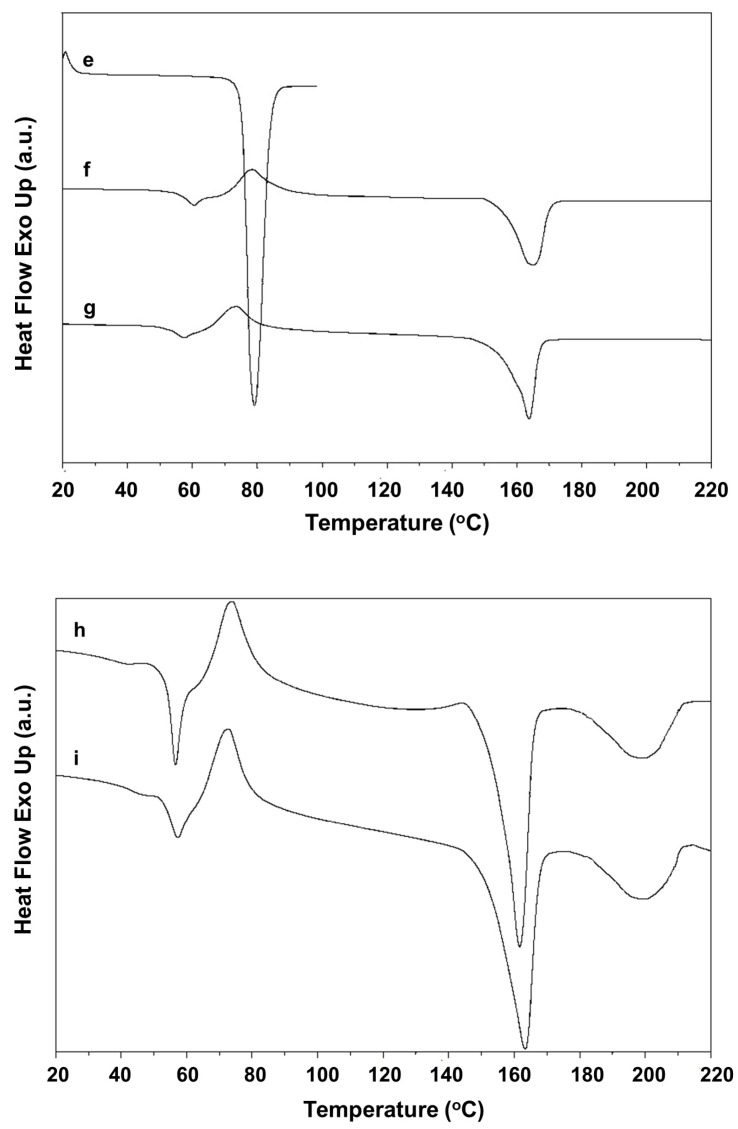
DSC thermograms of (a) RA powder, (b) PVA mat, (c) PVA/Ch mat, (d) PVA/Ch/RA mat, (e) LHC powder, (f) PLA mat, (g) PLA/LHC mat, (h) (PLA + PVA/Ch/RA) composite mat, and (i) (PLA/LHC + PVA/Ch/RA) composite mat.

The thermal behavior of the fibrous materials containing RA and/or LHC was evaluated by DSC (Figure 7). Figure 7a–d presents the thermograms of PVA, PVA/Ch, and PVA/Ch/RA mats and RA powder. The thermogram of RA showed a typical sharp endothermic melting peak at 169.3 °C (Figure 7a). The PVA mat displayed a glass transition temperature (T_g_) at 42 °C and a melting temperature (T_m_) at 205 °C. In this case, the determined degree of crystallinity was 43%. A broad endothermic peak in the range of 110 to 150 °C was detected in the thermal curves of PVA, PVA/Ch, and PVA/Ch/RA mats, which was most likely due to desorption of acetic acid from PVA and Ch. It was suggested that the observed decrease in T_g_ and T_m_ of PVA to 29 °C and 204 °C, respectively, in the PVA/Ch mats, was due to intermolecular interaction of PVA and Ch. The presence of Ch in the PVA/Ch mats led to a decrease in the degree of crystallinity of PVA (37%, Supplementary Materials, Table S3). The registered decrease in T_g_, T_m,_ and crystallinity degree of PVA is in accordance with data obtained by other authors for electrospun PVA/Ch materials [61]. The incorporation of RA in the PVA/Ch mats resulted in a shift of the peak corresponding to the T_m_ of PVA to a lower temperature, at 200 °C (Figure 7d). The degree of crystallinity of PVA in the PVA/Ch/RA mats (34%) was lower than that of the neat PVA mats (43%) as well as that of the PVA/Ch mat (37%) (Supplementary Materials, Table S3). The observed change in the thermogram of the PVA/Ch mats upon loading with RA might be explained by the interaction between the polymer partners and RA. Furthermore, no T_m_ for RA was detected in the thermograms of PVA/Ch/RA mats, indicating that RA incorporated in the fibers was in an amorphous state.

Figure 7f–i shows the DSC thermograms of the PLA, PLA/LHC, (PLA + PVA/Ch/RA), and (PLA/LHC + PVA/Ch/RA) mats. As seen in Figure 7e, the melting point of LHC is at 79 °C. In the DSC thermogram of the PLA mat, the characteristics of the polymer T_g_, cold crystallization temperature (T_cc_), and T_m_ were observed, and their values are shown in Table S4 (Supplementary Materials). The determined crystallinity degree of PLA in the PLA mat was ca. 30%. The presence of LHC in PLA/LHC mats resulted in a slight decrease in T_g_, T_cc_, and T_m_ of PLA, and the degree of crystallinity of the polymer was slightly higher compared to that of the PLA mat (Supplementary Materials, Table S4). As seen in Figure 7g, the T_m_ of LHC was absent in the thermogram of the PLA/LHC mat. This is an indication that the local anesthetic was in an amorphous state in the fibrous material. In the thermograms of the (PLA + PVA/Ch/RA) and (PLA/LHC + PVA/Ch/RA) composite fibrous materials, in addition to the peaks for T_m_ and T_g_ of PVA, peaks for T_g_, T_cc_, and T_m_ of PLA were also detected (Figure 7h,i, Supplementary Materials, Table S4). No significant difference was observed in the thermal parameters and the degree of crystallinity of PVA in the PVA/Ch/RA mats and in the (PLA + PVA/Ch/RA) and (PLA/LHC + PVA/Ch/RA) composite mats (Supplementary Materials, Tables S2 and S3). The degree of crystallinity of PLA in (PLA + PVA/Ch/RA) and (PLA/LHC + PVA/Ch/RA) mats was 30% and 41%, respectively. These values were slightly higher than the degree of crystallinity of PLA in the PLA mat (29%) and in the PLA/LHC mat (34%). No melting peaks characteristic for RA and LHC were registered in the DSC curves of (PLA + PVA/Ch/RA) and (PLA/LHC + PVA/Ch/RA) mats. Therefore, RA and LHC incorporated in the composite mats were in an amorphous state.

### 3.5. XRD Analysis of the Fibrous Materials

The XRD patterns of PVA, PVA/Ch, PVA/Ch/RA, (PLA + PVA/Ch/RA), and (PLA/LHC + PVA/Ch/RA) mats, as well as RA powder and LHC powder, are presented in Figure S6 (Supplementary Materials). In the XRD pattern of PVA mats, a diffraction was observed at 2*θ* around 20° (Supplementary Materials, Figure S6A(a)), which corresponds to the (101) plane of the PVA crystals [62]. As seen from Figure S6A(b,c) (Supplementary Materials), this diffraction was also present in the XRD patterns of PVA/Ch and PVA/Ch/RA mats, but it was with lower intensity and was broader. This can be attributed to the decreased crystallinity of PVA in the PVA/Ch and PVA/Ch/RA mats due to the presence of Ch and RA in the fiber composition of the respective fibrous materials. These results are consistent with the data obtained by the DSC analyses. As seen from Figure S6 (Supplementary Materials), diffractions corresponding to the crystalline phase of RA (2*θ* = 13.6°, 14.9°, 19.4°, and 26.5°) [63] were lacking in the XRD patterns of the PVA/Ch/RA mats, as well as of the (PLA + PVA/Ch/RA) and (PLA/LHC + PVA/Ch/RA) composite fibrous mats. This indicated that RA loaded in these mats was in an amorphous state. The presence of an amorphous halo in the XRD spectra of PLA and PLA/LHC mats revealed that PLA was in an amorphous state in these mats (Supplementary Materials, Figure S6B(a,b)). A broadened diffraction with a maximum close to 2*θ* = 20° was observed in the XRD patterns of the (PLA + PVA/Ch/RA) and (PLA/LHC + PVA/Ch/RA) composite mats (Supplementary Materials, Figure S6B(c,d)). The registration of diffraction with a maximum at 2*θ* of ca. 20° might be attributed to the presence of a certain crystalline phase of PVA. For (PLA/LHC + PVA/Ch/RA) and PLA/LHC mats, no additional peaks corresponding to the LHC crystalline phase were detected (2*θ* = 14.3°, 16.5°, 19.2°, and 25.0°) (Supplementary Materials, Figure S6D). This suggests that the LHC embedded in the electrospun materials was in an amorphous phase. The observed amorphization of RA and LHC in the fibrous mats is attributed to the rapid evaporation of the solvents during the electrospinning process, which hinders the crystallization of the bioactive compound.

### 3.6. Water Contact Angle of the Fibrous Materials

**Figure 8:**
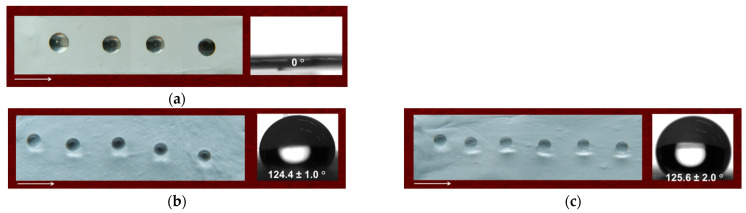

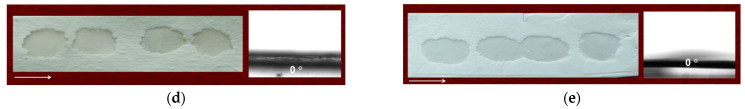
Digital photographs of water droplets deposited on the mat surface from (**a**) PVA/Ch/RA, (**b**) PLA, (**c**) PLA/LHC, (**d**) (PLA + PVA/Ch/RA), and (**e**) (PLA/LHC + PVA/Ch/RA). The white arrow indicates the collector rotation direction.

Since it is well known that the adhesion and proliferation of bacteria and fungi are affected by the hydrophilic/hydrophobic behavior of the surface of electrospun materials, it was of interest to determine their WCA. PVA/Ch/RA fibrous materials were found to be hydrophilic (Figure 8a).

The WCA value was 0°, and the water drop was immediately absorbed by the mat. As seen in Figure 8b,c, PLA and PLA/LHC mats were hydrophobic with WCA values of 124.4 ± 1.0° and 125.6 ± 2.0°, respectively. This was due to the hydrophobic behavior of PLA. For (PLA + PVA/Ch/RA) and (PLA/LHC + PVA/Ch/RA) composite fibrous materials, the presence of PVA/Ch/RA fibers led to hydrophilization of the surface of the materials. As seen from Figure 8d,e, the determined value of WCA was 0°. Furthermore, the wetted regions of these materials had an elliptical shape. This behavior, which had been observed in our previous studies and in the studies of other authors [64,65,66], might be explained by the fact that during its rapid absorption by the mat, the water drop migrated along the direction of fiber alignment. The major axis of the obtained elliptical wetted surface of the mat coincided with the fiber alignment direction. In our case, the direction of fiber arrangement aligned with the rotation direction of the rotating collector.

The observed hydrophilic behavior of the surface of the prepared PVA/Ch/RA materials and of the (PLA + PVA/Ch/RA) and (PLA/LHC + PVA/Ch/RA) composite fibrous materials is crucial for their future potential application in biomedical practice, e.g., as dressings for wound treatment, since the hydrophilicity of the materials is an important prerequisite for achieving an effective release, and therefore an adequate therapeutic effect, of the incorporated bioactive compounds. Moreover, it promotes the uptake of exudate from damaged tissue, which is essential for wound healing.

### 3.7. XPS Analysis of the Fibrous Materials

**Figure 9:**
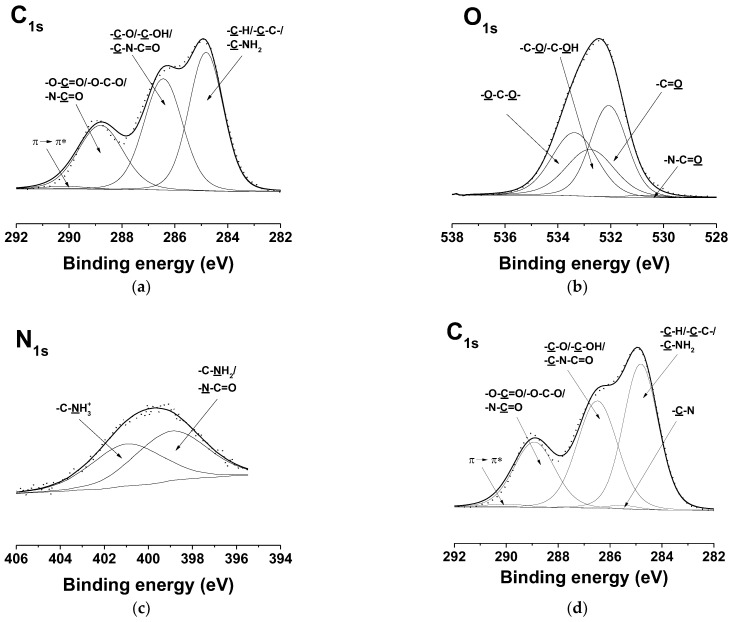

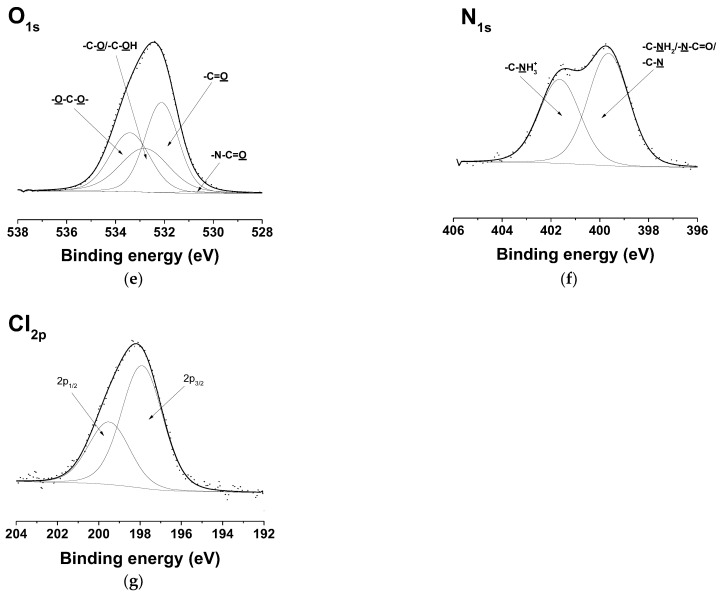
XPS peak fittings for (PLA + PVA/Ch/RA) mat [(**a**) C1s, (**b**) O1s, (**c**) N1s] and (PLA/LHC + PVA/Ch/RA) mat [(**d**) C1s, (**e**) O1s, (**f**) N1s, (**g**) Cl2p].

XPS was performed to analyze the surface composition of PVA/Ch/RA, (PLA + PVA/Ch/RA), and (PLA/LHC + PVA/Ch/RA) fibrous materials (Figure 9 and Supplementary Materials, Figure S7). For comparison, the surface composition of PLA and PLA/LHC mats was also studied by XPS. Four peaks were identified in the deconvoluted C_1s_ spectrum of the PVA/Ch/RA mats (Supplementary Materials, Figure S7a). Three of these peaks were at 285.0 eV, attributed to –C-H or –C-C- of PVA, Ch, and RA, and to –C-NH_2_ of Ch, at 286.4 eV—assigned to -C-O or -C-OH of PVA and Ch, to –C-N-C=O of Ch, and to -C-O or -C-OH of RA; at 288.5 eV—for -O-C=O of PVA and RA for -O-C-O- and -N-C=O of Ch. The fourth peak for the π→ π* shake-up satellite characteristic of the RA aromatic ring was detected at 290.3 eV. In the O_1s_ spectrum of the same mats, peaks at 533.2 eV assigned to -O-C-O- of Ch, at 532.6 eV assigned to -C-O or -C-OH of PVA and RA, and to -C-OH of Ch, and at 532.0 eV assigned to -O-C=O or -C=O of PVA and RA were observed (Supplementary Materials, Figure S7b). A peak of low intensity characteristic of -N-C=O from Ch was also recorded at 530.6 eV. An N_1s_ peak having two components—at 399.6 eV, assigned to the –N–C=O and –C–NH_2_ groups of Ch, and at 401.6 eV ascribed to the protonated amino groups (-NH_3_^+^) of Ch—was also registered (Supplementary Materials, Figure S7c). These peaks are consistent with previously reported values in the literature and correspond well to the structure of the PVA [67] and Ch components [68] present in the mats.

In the C_1s_ spectrum of the PLA mats (Supplementary Materials, Figure S8a), the presence of three peaks was detected: at 285.0 eV, assigned to –C-H or –C-C-; at 286.9 eV, for -C-O; and at 288.9 eV, for -O-C=O [69]. Two peaks were identified in the O_1s_ spectrum (Supplementary Materials, Figure S8b): at 533.6 eV ascribed to -C-O and at 532.2 eV corresponding to -O-C=O. Compared to the detailed C_1s_ spectrum of the neat PLA fibers (Supplementary Materials, Figure S8a), two new peaks at 285.9 eV, attributed to -C-N from LHC, and at 290.3 eV, corresponding to the π→ π* shake-up satellite ascribed to the LHC aromatic ring, were detected in the spectrum of PLA/LHC fibers (Supplementary Materials, Figure S8c) [70]. The detailed O_1s_ spectrum (Supplementary Materials, Figure S8d) showed two peaks—a peak at 533.6 eV for -C-O from PLA and a peak at 532.3 eV for -O-C=O from PLA and for -C=O from LHC. A peak assigned to the nitrogen atoms from -C-N of the incorporated LHC at a binding energy of 400.5 eV (Supplementary Materials, Figure S8e) also appeared [70], as well as a Cl_2p_ peak at 198.1 eV (Cl_2p3/2_) and at 199.7 eV (Cl_2p1/2_) from the incorporated LHC (Supplementary Materials, Figure S8f).

In the XPS detailed C_1s_ spectra of (PLA + PVA/Ch/RA) composite fibrous materials, four peaks were recorded (Figure 9a). Two of these peaks were at 285.0 eV, attributed to –C-H or –C-C- of PLA, PVA, Ch, and RA, and to –C-NH_2_ of Ch, and at 286.4 eV—characteristic of –C-O of PLA, -C-O or –C-OH of PVA, Ch, and RA, and of –C-N-C=O of Ch. In the detailed C_1s_ spectra of (PLA + PVA/Ch/RA) mats compared to those of PVA/Ch/RA mats, the peak at 288.8 eV increased in intensity, as it was related not only to –O-C=O of PVA and RA and to -O-C-O- and -N-C=O of Ch, but also to –O-C=O of PLA. The presence of RA in the composite fibers was also indicated by the appearance of a peak at 290.3 eV corresponding to a π→ π* shake-up satellite, characteristic of the benzene ring of RA. In the detailed O_1s_ spectra of the same composite mats (Figure 9b) compared to those of PLA mats (Supplementary Materials, Figure S8b), in addition to the peaks at 532.1 eV assigned to -O-C=O or -C=O of PLA, PVA, and RA and at 533.4 eV assigned to -O-C-O- of PLA and Ch, new peaks were observed at 532.8 eV for -C-O or -C-OH of PVA and RA and for -C-OH of Ch as well as at 530.6 eV for -N-C=O of Ch. In comparison with the XPS spectra of PLA mats (Supplementary Materials, Figure S8a,b), in the spectra of the (PLA + PVA/Ch/RA) composite mats, the appearance of an N_1s_ peak of two components (at 399.2 eV for –N–C=O and for –C–NH_2_ groups of Ch and at 401.1 eV for -NH_3_^+^ of Ch) was recorded (Figure 9c).

In the detailed C_1s_ spectra of the (PLA/LHC + PVA/Ch/RA) composite mats compared to those of the (PLA + PVA/Ch/RA) composite mats, the appearance of a new peak was identified at 285.9 eV due to –C-N of the incorporated LHC (Figure 9a,d). A peak at 290.3 eV was also observed for the π→ π* shake-up satellite characteristic of the RA and LHC rings. The O_1s_ spectra of the (PLA/LHC + PVA/Ch/RA) mats, similar to those from (PLA + PVA/Ch/RA) mats, consisted of four peaks—at 533.4 eV for -O-C-O- of Ch and of PLA, at 532.8 eV for -C-O or -C-OH of PVA and RA, and for -C-OH of Ch, at 532.1 eV for -O-C=O or -C=O from PVA, RA, and PLA and for -C=O from LHC, as well as a peak at 530.6 eV for -N-C=O from Ch (Figure 9b,e). An N_1s_ peak consisting of two components—at 399.6 eV for –N–C=O and –C–NH_2_ groups from Ch and for –C–N from LHC and at 401.6 eV for –NH_3_^+^ from Ch—was recorded (Figure 9f). The presence of a Cl_2p_ peak at 198.0 eV (Cl_2p3/2_) and at 199.6 eV (Cl_2p1/2_) due to the incorporation of LHC in the composite fibers was also observed (Figure 9g).

The results obtained from XPS analyses confirmed the presence of RA and/or LHC on the surface of PVA/Ch/RA mats, as well as on the (PLA + PVA/Ch/RA) and (PLA/LHC + PVA/Ch/RA) composite mats.

### 3.8. Tensile Tests of the Fibrous Materials

To evaluate the mechanical behavior of the novel PVA/Ch/RA, (PLA + PVA/Ch/RA), and (PLA/LHC + PVA/Ch/RA) fibrous materials, tensile tests were carried out. PVA/Ch, PLA, and PLA/LHC mats were used as control fibrous materials. The determined values of tensile strength, Young’s modulus, and elongation at break of the studied fibrous materials are presented in Table 1. As seen, PVA/Ch mats had a tensile strength of 7.30 ± 0.60 MPa, a Young’s modulus of 112.90 ± 27.40 MPa, and an elongation at break of around 40%.

**Figure 10:**
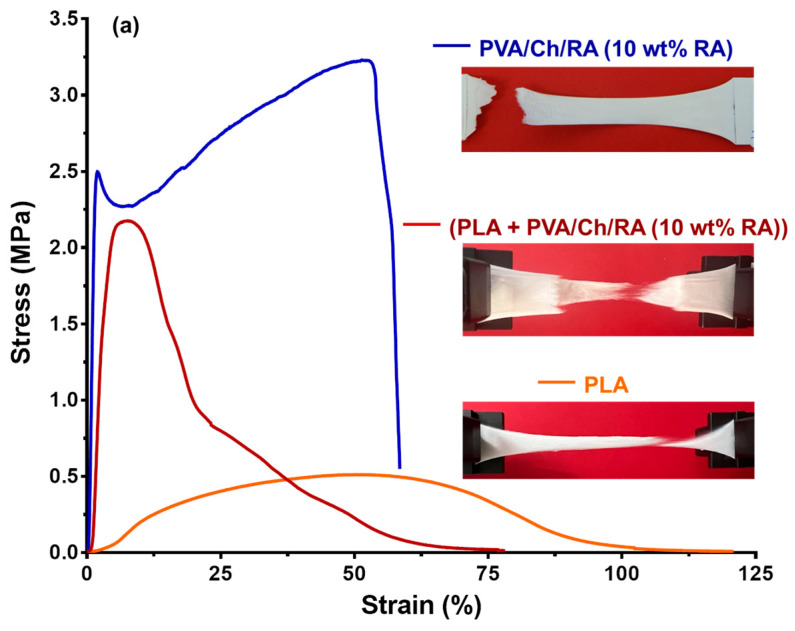

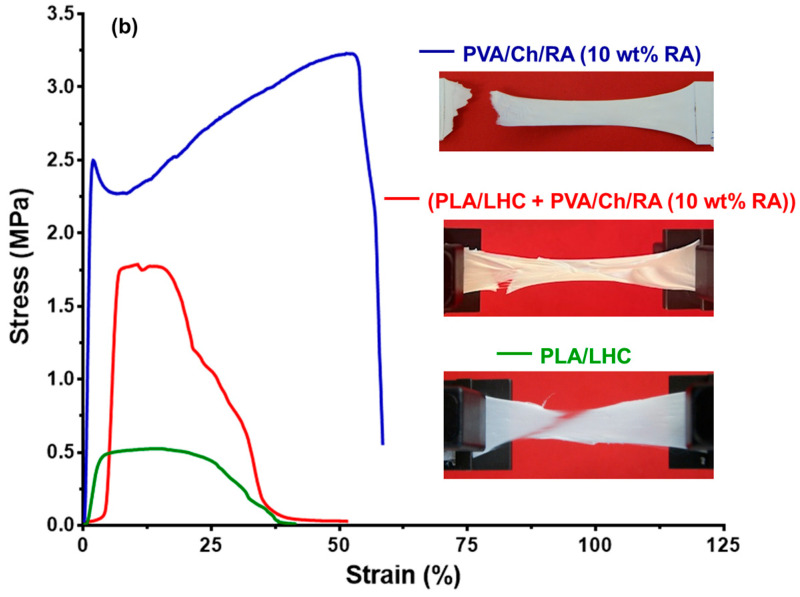
Stress–strain curves of: (**a**) PVA/Ch/RA (10 wt% RA), (PLA + PVA/Ch/RA (10 wt% RA)), and PLA mats, and (**b**) PVA/Ch/RA (10 wt% RA), (PLA/LHC + PVA/Ch/RA (10 wt% RA)) and PLA/LHC mats. The number of the tested specimens of each fibrous material was 10.

Incorporation of RA into PVA/Ch mats resulted in a slight decrease in tensile strength (3.20 ± 0.03 MPa), as well as an increase in Young’s modulus (246.40 ± 2.10 MPa) and elongation at break (58.60 ± 0.20%). The decrease in tensile strength and increase in elongation at break can be attributed to the lower degree of crystallinity of PVA in PVA/Ch/RA mats (33.7%) compared to that in PVA/Ch mats (37.0%). The registered higher value of Young’s modulus was most likely due to the presence of hydrogen bonds between the polymers and RA, similarly to other electrospun materials composed of a polymer and a polyphenolic compound [71,72]. Figure 10a compares the stress–strain curve for (PLA + PVA/Ch/RA) mats with those of the constituent fibers of the composite fibrous material, namely PLA and PVA/Ch/RA. As seen from the presented curve for PLA mats and from the data shown in Table 1, PLA fibrous materials had low tensile strength and a high elongation at break value. These properties of PLA mats were most probably attributed to the fact that PLA was in an amorphous state in the fibers, as evidenced by DSC and XRD analyses. Necking was observed during tensile testing of the samples. When comparing the mechanical parameters of PVA/Ch/RA, (PLA + PVA/Ch/RA), and PLA mats (Figure 10a; Table 1), it is evident that the (PLA + PVA/Ch/RA) composite fibrous materials had values of tensile parameters that were between the values characteristic for PLA and PVA/Ch/RA mats. These findings clearly demonstrate that the composite material was made of both types of fibers, PLA and PVA/Ch/RA, respectively. During the tensile testing of the composite mats, non-uniform breaking of the constituent fibers was detected (Figure 10a). The registration of the fibers in which necking was observed could be attributed to those made of PLA. As seen from Table 1 and from Figure 10b, the incorporation of LHC into PLA mats led to an insignificant change in the tensile strength value (0.53 ± 0.05 MPa), an increase in Young’s modulus (26.70 ± 9.30 MPa), and a decrease in the elongation at break (39.80 ± 2.60%). The observed increase in Young’s modulus and decrease in elongation at break could be due to the greater degree of crystallinity of PLA in the PLA/LHC mat (34%) compared to the PLA mat (29%), as determined by DSC analysis.

The fact that (PLA/LHC + PVA/Ch/RA) composite fibrous materials demonstrated mechanical characteristics with values intermediate between those of PLA/LHC and PVA/Ch/RA fibers (Table 1 and Figure 10b) provided strong evidence for the contribution of both fiber types to the composite structure. For (PLA/LHC + PVA/Ch/RA) mats, the sample broke during the tensile tests without undergoing necking.

It is known that in view of the potential applications of polymeric materials as wound dressings, it is important that their mechanical properties are close to those of human skin, namely, a tensile strength from 1 to 32 MPa, a Young’s modulus from 2.9 to 150 MPa, and an elongation at break from 17% to 207% [73]. The results obtained from tensile tests for the PVA/Ch/RA, (PLA + PVA/Ch/RA), and (PLA/LHC + PVA/Ch/RA) mats showed that novel fibrous materials exhibited parameters within the range of human skin, making them suitable for use as atraumatic wound dressings.

### 3.9. In Vitro Release Studies

**Figure 11:**
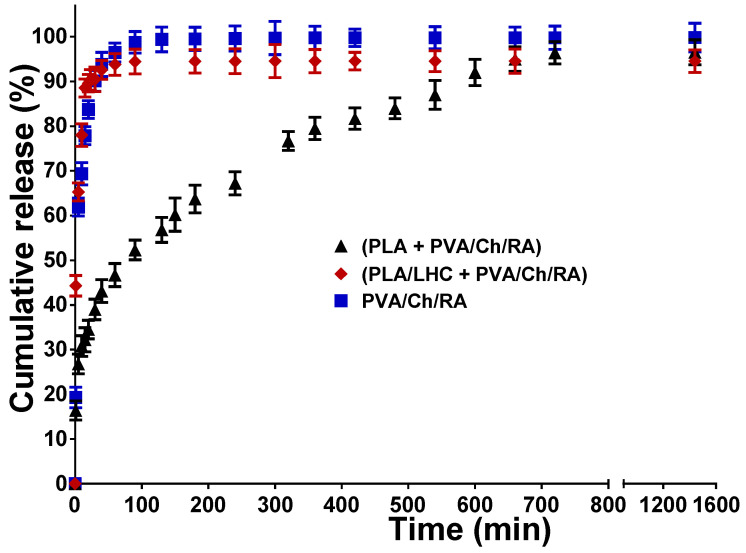
In vitro study of the RA release from mats: PVA/Ch/RA (10 wt% RA), (PLA + PVA/Ch/RA (10 wt% RA)), and (PLA/LHC (10 wt% LHC) + PVA/Ch/RA (10 wt% RA)). PBS; 37 °C; pH: 7.4; ionic strength: 0.1.

In vitro release studies were conducted UV spectrophotometrically using PBS buffer of pH 7.4 at 37 °C. Figure 11 displays the RA release profiles from the PVA/Ch/RA mats and from the (PLA + PVA/Ch/RA) and (PLA/LHC + PVA/Ch/RA) composite mats.

In the case of PVA/Ch/RA and (PLA/LHC + PVA/Ch/RA) mats, a rapid release of RA was recorded, and in the initial 20 min the amount of RA released from the PVA/Ch/RA and (PLA/LHC + PVA/Ch/RA) mats was 83.7% and 89.6%, respectively. For PVA/Ch/RA and (PLA/LHC + PVA/Ch/RA) mats, the amount of released RA was ca. 99.8% and 94.6% in 1440 min, respectively. In the case of (PLA + PVA/Ch/RA) composite fibrous materials, a first stage of rapid release of RA (up to ca. 20 min) was recorded, followed by a gradual release stage (Figure 11). During the first stage, only ca. 35% of the RA incorporated in the mats was released. A gradual release of RA followed in the interval from 20 to 720 min, reaching ca. 96.5%. To explain the observed difference in the release profiles of RA from the respective composite mats, we studied the variation of the swelling degree of (PLA + PVA/Ch/RA) and (PLA/LHC + PVA/Ch/RA) composite mats measured in PBS buffer of pH 7.4 at 37 °C as a function of time (Supplementary Materials, Figure S9). As seen from Figure S9 (Supplementary Materials), (PLA + PVA/Ch/RA) composite mats swelled more slowly than the (PLA/LHC + PVA/Ch/RA) mats. The equilibrium swelling degree of (PLA/LHC + PVA/Ch/RA) mats (475.0 ± 11.8%) was higher than that of (PLA + PVA/Ch/RA) mats (386.3 ± 12.2%). Therefore, it might be assumed that the observed slower release of RA from (PLA + PVA/Ch/RA) mats compared to that from (PLA/LHC + PVA/Ch/RA) mats is most likely due to the slower swelling of (PLA + PVA/Ch/RA) mats in the PBS buffer at 37 °C. Furthermore, all studied fibrous materials are hydrophilic, and the incorporated RA is in an amorphous state, which favors the release of RA into the buffer.

The in vitro release profile of LHC from the respective fibrous materials studied in PBS solution is presented in Figure S10 (Supplementary Materials). In the case of the PLA/LHC mats, ca. 35% of LHC was released in the first 15 min. For the (PLA/LHC + PVA/Ch/RA) composite mats, the release was faster, with the amount of LHC released in 15 min being ca. 82.8%. The amount of LHC released from the PLA/LHC and (PLA/LHC + PVA/Ch/RA) mats in 1440 min was approximately 95.0% and 94.6%, respectively. The observed difference in the LHC release profiles could be attributed to the difference in the hydrophilic-hydrophobic properties of the mats. The determined WCA value for PLA/LHC mats was 125.6 ± 2.0°, while the WCA for (PLA/LHC + PVA/Ch/RA) composite fibrous materials was 0°. The hydrophilic behavior of the (PLA/LHC + PVA/Ch/RA) fibrous materials favors the penetration of water molecules into the structure of the mats, thereby enhancing the release rate of the bioactive compounds. The evidence of the XRD amorphous state of LHC loaded in the fibrous materials facilitates the release of this bioactive compound.

To better elucidate the release kinetics of RA and LHC from the corresponding mats, the release data were analyzed using the Korsmeyer–Peppas model [41]. In this model, a plot of log(M_t_/M_∞_) against log(t) provides information about the release mechanism, with the slope of the fitted line corresponding to the release exponent (n). As presented in Figures S11 and S12 (Supplementary Materials), all three systems exhibited an excellent linear fit, with correlation coefficients (R^2^) in the range of 0.90–0.99, indicating the suitability of the model for describing their release behavior. The calculated release exponents for the RA-loaded mats, namely, PVA/Ch/RA, (PLA + PVA/Ch/RA), and (PLA/LHC + PVA/Ch/RA), were 0.42, 0.25, and 0.24, respectively (Supplementary Materials, Figure S11). In addition, the values of the release exponent (n) for PLA/LHC and (PLA/LHC + PVA/Ch/RA) mats were 0.44 and 0.21, respectively (Supplementary Materials, Figure S12). These values fall below the threshold of 0.45, which is characteristic of a Fickian diffusion-controlled process. This suggests that the release of the bioactive compounds from the studied mats was predominantly governed by diffusion through the polymeric network. Thus, the release of RA and LHC from the prepared materials can be consistently described as diffusion-driven, which is an important consideration for predicting and controlling their behavior in potential biomedical applications. The findings obtained from this study are in good agreement with published studies on drug-loaded PVA/Ch- or PVA-based materials [74,75].

### 3.10. Antioxidant Activity of Fibrous Materials

Currently, one of the main strategies in the design of novel polymer materials for wound healing is the incorporation of a biologically active agent with antioxidant properties [76]. The goal is to prevent oxidative damage to the tissue and support tissue regeneration [77].

Ch, RA, and LHC have been reported to possess antioxidant properties [78,79,80]. A DPPH^•^ scavenging assay was carried out to evaluate the antioxidant activity of PVA/Ch/RA, (PLA + PVA/Ch/RA), and (PLA/LHC + PVA/Ch/RA) fibrous mats.

**Figure 12:**
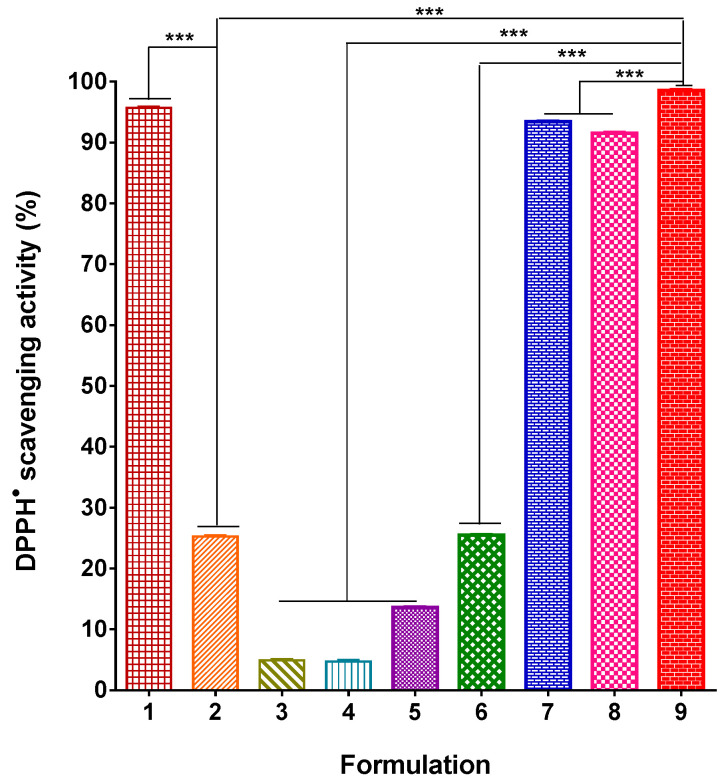
DPPH^•^ scavenging activity of 1—RA solution; 2—LHC solution; 3—PLA mat; 4—PVA mat; 5—PVA/Ch mat; 6—PLA/LHC mat; 7—PVA/Ch/RA mat; 8—(PLA + PVA/Ch/RA) mat; and 9—(PLA/LHC + PVA/Ch/RA) mat. *** *p* < 0.001 (Table S5, Supplementary Materials).

The antioxidant activity of PVA and PVA/Ch mats, not containing RA, as well as PLA and PLA/LHC mats, was also studied. As seen from Figure 12, PVA and PLA mats are characterized by very low activity. In these cases, the absorbance of DPPH^•^ decreased by 4.70 ± 0.19% and by 4.91 ± 0.08% for the PVA and PLA mats, respectively. After 30 min of contact of the DPPH^•^ solution with PVA/Ch mats, the absorbance of DPPH^•^ fell slightly by 13.6%. In contrast, the obtained RA-containing fibrous materials manifested high antioxidant activity close to that of the individual bioactive compound RA. In these cases, the absorbance of DPPH^•^ decreased significantly—by 93.42 ± 0.06%, 91.5 ± 0.13%, and 95.7 ± 0.06%, respectively, for PVA/Ch/RA mats, (PLA + PVA/Ch/RA) mats, and free RA solution. This indicated that RA incorporated in the PVA/Ch/RA and (PLA + PVA/Ch/RA) mats imparted them good antioxidant activity. The PLA/LHC mat exhibited a weak DPPH^•^ scavenging ability (approximately 25.5%), which was close to that of the free LHC solution (Figure 12). Mats containing RA and LHC [(PLA/LHC + PVA/Ch/RA) mats], prepared by dual spinneret electrospinning, possessed higher antioxidant activity (approximately 98.5%) than PVA/Ch/RA, (PLA/LHC + PVA/Ch/RA), and PLA/LHC mats (Figure 12). Most likely, the combination of the antioxidant activities of LHC and RA incorporated in the fibrous materials [(PLA/LHC + PVA/Ch/RA) mats] led to an increase in the total antioxidant activity of these materials.

### 3.11. Evaluation of the Antibacterial and Antifungal Activity of the Fibrous Materials

**Figure 13:**
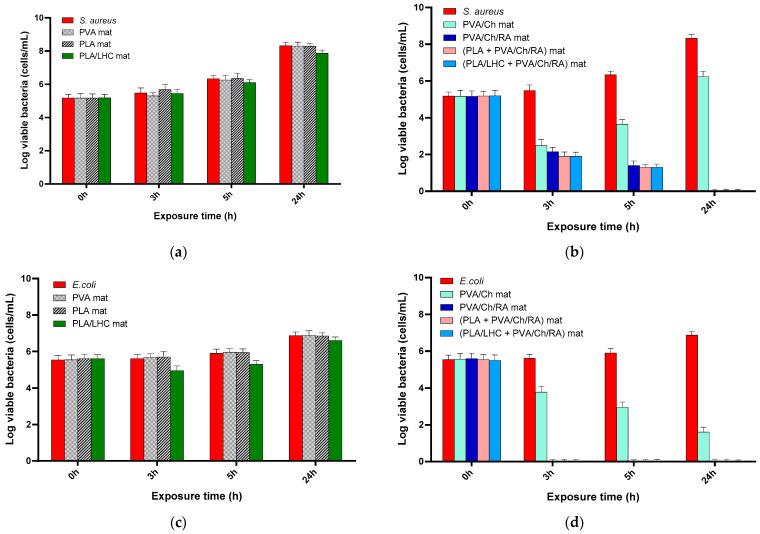

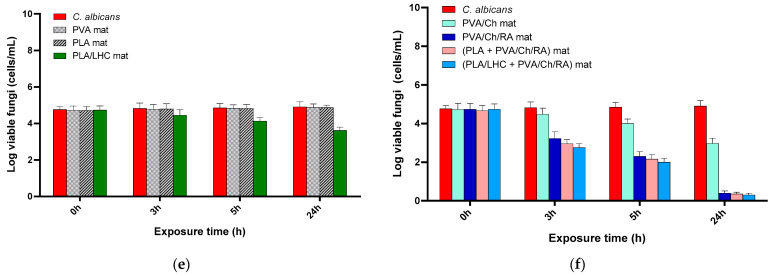
Logarithmic plot of the viable bacteria cell number versus the exposure time for control—bacteria cell suspension, PVA mat, PLA mat, PLA/LHC mat, PVA/Ch mat, PVA/Ch/RA mat, (PLA + PVA/Ch/RA) mat, and (PLA/LHC + PVA/Ch/RA) mat. Tests have been carried out against (**a**,**b**) *S. aureus*, (**c**,**d**) *E. coli*, and (**e**,**f**) *C. albicans*. Data represent the mean ± standard deviation (n = 3). The RA concentration was 1500 μg/mL in (**b**,**d**) and 3000 μg/mL in (**f**). The Ch concentration was 714 μg/mL in (**b**,**d**), and 1428 μg/mL in (**f**). The LHC concentration was 3000 μg/mL in (**a**–**d**), and 6000 μg/mL in (**e**,**f**).

The antibacterial and antifungal activity of fibrous mats containing RA, as well as those containing RA and LHC, against the bacteria *S. aureus* and *E. coli* and the fungi *C. albicans*, was tested by counting the viable bacteria and fungi that remained in the suspension after incubating the mats for 3, 5, and 24 h. The number of surviving bacteria or fungi was determined by plating and counting CFUs in a solid medium. The antibacterial and antifungal activities of PVA, PVA/Ch, PLA, and PLA/LHC mats were also evaluated. PVA and PLA mat controls did not suppress the growth of the *S. aureus* bacteria, and log(CFU/mL) was approximately 8.3 at 24 h (Figure 13a). This value was close to that of *S. aureus* control over the same time period. In the case of the PLA/LHC mats, a negligible change in the number of viable *S. aureus* cells (log(CFU/mL) was 7.9) was observed after 24 h contact (Figure 13a). For PVA/Ch mats with Ch content of 714 μg/mL the *S. aureus* titer reduction was up to 2.48 ± 0.34 log for a contact time of 3 h, but for 24 h the *S. aureus* titer reduction was only 6.24 ± 0.29 log (Figure 13b). In contrast, RA-loaded PVA/Ch mats with Ch content—714 μg/mL and RA content—1500 μg/mL killed all *S. aureus* bacteria within a contact time of 24 h (Figure 13b). A similar bactericidal effect against *S. aureus* was also exhibited by the (PLA + PVA/Ch/RA) and (PLA/LHC + PVA/Ch/RA) mats for the same contact time.

PVA/Ch/RA, (PLA + PVA/Ch/RA), and (PLA/LHC + PVA/Ch/RA) fibrous materials were found to inhibit the growth of Gram-negative bacteria *E. coli* faster than that of Gram-positive bacteria *S. aureus* at the same concentration of Ch—714 μg/mL, RA—1500 μg/mL, and LHC—3000 μg/mL (Figure 13b,d). In the case of these fibrous materials, a reduction of the bacterial titer to zero was detected within a contact time of 3 h (Figure 13d). At the same contact time, the PVA/Ch and PLA/LHC mats decreased the number of viable cells to 3.8 and 5.0 log, respectively (Figure 13c,d).

The results from the microbiological screening showed that PVA/Ch/RA, (PLA + PVA/Ch/RA), and (PLA/LHC + PVA/Ch/RA) mats displayed weaker activity against the fungi *C. albicans* than against the bacteria *E. coli* and *S. aureus* (Figure 13). At a content of Ch—1428 μg/mL, RA—3000 μg/mL, and LHC—6000 μg/mL, the PVA/Ch/RA, (PLA + PVA/Ch/RA), and (PLA/LHC + PVA/Ch/RA) mats during a contact time of 24 h led to a decrease in the titer of *C. albicans* by 0.4, 0.35, and 0.3 log, respectively (Figure 13f).

In comparison, over the same time period, the PVA/Ch and PLA/LHC mats caused only 2.97 and 3.62 log reductions in fungal titers, respectively (Figure 13e,f). In contrast, PVA and PLA mats alone were unable to suppress the growth of *E. coli* and *C. albicans* (Figure 13c,e). The obtained results showed that the observed antibacterial and antifungal activity of PVA/Ch/RA, (PLA + PVA/Ch/RA), and (PLA/LHC + PVA/Ch/RA) mats is mainly due to the presence of RA and Ch, which possess high antibacterial and antifungal efficacy.

The adhesion of pathogenic *S. aureus* cells on the surface of the fibrous materials after 24 h of contact of the latter with a bacterial cell suspension was investigated by SEM. As seen in Figure S13a (Supplementary Materials), a large number of cells adhered to the surface of the hydrophobic PLA mat. Only single bacteria or no bacteria were detected on the surface of the (PLA + PVA/Ch/RA) and (PLA/LHC + PVA/Ch/RA) mats (Supplementary Materials, Figure S13b,c). This tendency to inhibit the adhesion of bacteria and their proliferation is most likely due to the presence of RA and Ch, which exhibited high antibacterial activity. These materials are promising for application in the biomedical field, e.g., as materials having a surface capable of killing pathogenic bacteria.

## 4. Conclusions

Herein, for the first time, PVA/Ch/RA fibrous materials were prepared by one-step electrospinning of a blend solution of the components. The simultaneous dual electrospinning of a PVA/Ch/RA solution and a PLA or PLA/LHC solution enabled the fabrication of novel multifunctional fibrous materials consisting of PVA/Ch/RA fibers and PLA or PLA/LHC fibers, as evidenced by CLSM. DSC and XRD analyses revealed that the RA and LHC incorporated in the innovative materials were in an amorphous state. The appropriate selection of composition and the developed strategies for preparing materials with diverse, targeted architectures allowed the modulation of their hydrophilic/hydrophobic characteristics and the in vitro release behavior of the incorporated RA and LHC. The composite fibrous materials obtained by dual spinneret electrospinning exhibited good tensile properties, with values intermediate between those of the constituent fibers, indicating the contribution of both fiber types to the composite structure. Furthermore, the mechanical properties of the materials fell within the range typical for human skin, supporting their potential application as wound-healing dressings. It was shown that the presence of hydrophobic fibers of PLA or PLA/LHC in the composite fibrous materials imparted structural stability to them in a physiological medium. PVA/Ch/RA and (PLA + PVA/Ch/RA) fibrous materials were found to manifest high antioxidant activity (93.42 ± 0.06 and 91.50 ± 0.13%, respectively), close to that of RA (95.70 ± 0.06%). An increase in the antioxidant activity (ca. 98.5%) of the fibrous materials was observed when both RA and LHC were incorporated into them. Microbiological tests showed that the (PLA + PVA/Ch/RA) and (PLA/LHC + PVA/Ch/RA) composite fibrous materials exhibited significant antibacterial and antifungal activity against the pathogenic bacteria *S. aureus* and *E. coli* and *C. albicans* fungi. These materials had the ability to inhibit the adhesion of the bacteria *S. aureus*. The combination of antioxidant and antimicrobial properties, in conjunction with the favorable mechanical characteristics of the composite fibrous materials, makes this novel non-woven textile a promising candidate for applications as active textile materials in medicine and medical skincare. Potential uses include antibacterial and atraumatic wound dressings, as well as systems for local drug delivery.

## Figures and Tables

**Table 1:** Tensile properties of the electrospun fibrous materials.

Fibrous Materials	Tensile Strength, MPa	Young’s Modulus, MPa	Elongation at Break, %
PVA/Ch	7.30 ± 0.60	112.90 ± 27.40	40.40 ± 3.50
PVA/Ch/RA (10 wt% RA)	3.20 ± 0.03	246.40 ± 2.10	58.60 ± 0.20
(PLA + PVA/Ch/RA (10 wt% RA))	2.20 ± 0.18	121.00 ± 28.90	81.50 ± 6.90
(PLA/LHC + PVA/Ch/RA (10 wt% RA))	1.80 ± 0.40	86.00 ± 28.90	51.80 ± 25.20
PLA/LHC	0.53 ± 0.05	26.70 ± 9.30	39.80 ± 2.60
PLA	0.52 ± 0.07	3.60 ± 0.60	115.60 ± 11.70

## Data Availability

The data that support the findings of this study are available from the corresponding author upon request.

## Supplementary Materials

The following supporting information can be downloaded at https://www.mdpi.com/article/10.3390/polym17192657/s1, Scheme S1: Schematic illustration of the dual spinneret electrospinning setup used for preparation of (PLA + PVA/Ch/RA) and (PLA/LHC + PVA/Ch/RA) multifunctional fibrous materials: (1) PLA or PLA/LHC solution labeled with fluorescein; (2) PVA/Ch/RA solution; Figure S1: CLSM images of PVA/Ch/RA (10 wt%) fibers. PVA/Ch/RA fibers are stained in blue by the embedded RA. Selected images of Z-layers at a depth of 3 µm (a), 5 µm (b), and 10 µm (c). The Z step of the images is 1 μm. Scale bars = 10 µm. The branching of some of the main fibers is indicated with arrows, and the spindle-like defects are marked with dotted lines.; Figure S2: CLSM images of PLA/LHC/fluorescein (0.5 wt%) fibers. PLA/LHC/fluorescein fibers are stained in green by the embedded fluorescein. Selected images of Z-layers at a depth of 3 µm (a), 5 µm (b), and 9 µm (c). The Z step of the images is 1 μm. Scale bars = 10 µm; Figure S3: SEM micrographs of the fabricated electrospun mats: (PLA + PVA/Ch/RA) after 24 h (a) and 48 h (b) immersion in PBS buffer; and (PLA/LHC + PVA/Ch/RA) after 24 h (c) and 48 h (d) immersion in PBS buffer; Figure S4: ATR–FTIR spectrum of RA (powder); Figure S5: ATR–FTIR spectrum of LHC (powder); Figure S6: XRD patterns of: (A) (a) PVA, (b) PVA/Ch, (c) PVA/Ch/RA (10 wt%); (B) (a) PLA, (b) PLA/LHC, (c) (PLA + PVA/Ch/RA), (d) (PLA/LHC + PVA/Ch/RA), (C) RA powder, and (D) LHC powder; Figure S7: XPS peak fittings for PVA/Ch/RA (10wt% RA) mat [(a) C1s, (b) O1s, (c) N1s]; Figure S8: XPS peak fittings for PLA mat [(a) C1s, (b) O1s] and PLA/LHC mat [(c) C1s, (d) O1s, (e) N1s, (f) Cl2p]; Figure S9: Equilibrium swelling degree (α_eq_), of (PLA + PVA/Ch/RA) mat and of (PLA/LHC + PVA/Ch/RA) mat in PBS (pH 7.4) at 37 °C, versus time; Figure S10: In vitro study of the LHC release from mats: PLA/LHC (10 wt% LHC) and (PLA/LHC (10 wt% LHC) + PVA/Ch/RA (10 wt% RA)); PBS, 37 °C, pH 7.4, ionic strength, 0.1; Figure S11: Korsmeyer–Peppas model of RA release from mats: PVA/Ch/RA (10 wt% RA) (a), (PLA + PVA/Ch/RA (10 wt% RA)) (b), and (PLA/LHC (10 wt% LHC) + PVA/Ch/RA (10 wt% RA)) (c) in PBS at 37 °C, pH 7.4, ionic strength 0.1; Figure S12: Korsmeyer–Peppas model of LHC release from mats: PLA/LHC (10 wt% LHC) (a) and (PLA/LHC (10 wt% LHC) + PVA/Ch/RA (10 wt% RA)) (b) in PBS at 37 °C, pH 7.4, ionic strength 0.1; Figure S13: SEM micrographs of fibrous materials that have been incubated in *S. aureus* cell culture (10^7^ cells/mL) for 24 h at 37 °C, (a) PLA, (b) (PLA + PVA/Ch/RA), and (c) (PLA/LHC + PVA/Ch/RA); magnification ×4000; Table S1: Concentration of the components for preparation of the solutions subjected to simultaneous dual spinneret electrospinning for fabrication of the multifunctional composite fibrous materials; Table S2: Dynamic viscosity (η) and conductivity (σ) of the spinning solutions and average fiber diameter of the electrospun mats; Table S3: Thermal characteristics of the mats and degree of crystallinity of PVA; Table S4: Thermal characteristics of the mats and degree of crystallinity of PVA and PLA; Table S5: Results from the one-way analysis of variance (ANOVA) utilized to assess the statistical significance of the data presented in Figure 12 of the manuscript using GraphPad PRISM. References [38,39] are cited in the Supplementary Materials.
