# Supplementary material for: Multifunctional Electrospun Materials from Poly(Vinyl Alcohol)/Chitosan and Polylactide Incorporating Rosmarinic Acid and Lidocaine with Antioxidant and Antimicrobial Properties
Source: Polymers (Basel). 2025 Sep 30;17(19):2657. doi: 10.3390/polym17192657 (PMC12526612; doi:10.3390/polym17192657)
Supplement: Supplementary file 1 [file polymers-17-02657-s001.zip › polymers-3884797-supplementary.pdf]

Supplementary Materials

Multifunctional Electrospun Materials from Poly(Vinyl Alcohol)/Chitosan and Polylactide Incorporating Rosmarinic Acid and Lidocaine with Antioxidant and Antimicrobial Properties

Milena Ignatova[1,*], Dilyana Paneva[1], Selin Kyuchyuk[1], Nevena Manolova[1], Iliya Rashkov[1,*], Milena Mourdjeva[2], Nadya Markova[3]

[1]Laboratory of Bioactive Polymers, Institute of Polymers, Bulgarian Academy of Sciences, Acad. G. Bonchev St., Bl. 103A, BG-1113 Sofia, Bulgaria; panevad@polymer.bas.bg (D.P.); selin.erdinch@polymer.bas.bg (S.K.); manolova@polymer.bas.bg (N.M.) (Nevena Manolova)
[2]Institute of Biology and Immunology of Reproduction "Acad. Kiril Bratanov", Bulgarian Academy of Sciences, 73, Tsarigradsko shose blvd., 1113 Sofia, Bulgaria; mourdjeva@ibir.bas.bg (M.M.)
[3]Institute of Microbiology, Bulgarian Academy of Sciences, Acad. G. Bonchev St., Bl. 26, BG-1113 Sofia, Bulgaria; nadya.markova@microbio.bas.bg (N.M.) (Nadya Markova)
* Correspondence: ignatova@polymer.bas.bg (M.I.); rashkov@polymer.bas.bg (I.R.); Tel: +359-(0)2-9792239 (M.I.)

Table S1. Concentration of the components for preparation of the solutions subjected to simultaneous dual spinneret electrospinning for fabrication of the multifunctional composite fibrous materials

**Fibrous materials composition**	**LHC-(non)containing PLA solution***		**PVA/Ch/RA solution****		
	PLA, % (w/w)	LHC, % (w/w)	PVA, % (w/w)	Ch, % (w/w)	RA, % (w/w)
(PLA + PVA/Ch/RA)	10	-	8.0	0.40	0.84
(PLA + PVA/Ch/RA)	9.9	0.99	8.0	0.40	0.84

* DCM/DMSO [75/25 (w/w)] mixed solvent system was used as a solvent; ** 30 % v/v acetic acid aqueous solution was used as a solvent.

Table S2. Dynamic viscosity (η) and conductivity (σ) of the spinning solutions, and average fiber diameter of the electrospun mats.

**Electrospun mats**	η (cP)	σ (μS/cm)	d (nm)
PVA	1530	1343	425 ± 60
PVA/Ch	1220	1455	210 ±60
PVA/Ch/RA (5 wt%)	1500	1470	270 ± 100
PVA/Ch/RA (7 wt%)	1550	1450	310 ±140
PVA/Ch/RA (10 wt%)	1500	1450	274 ±140

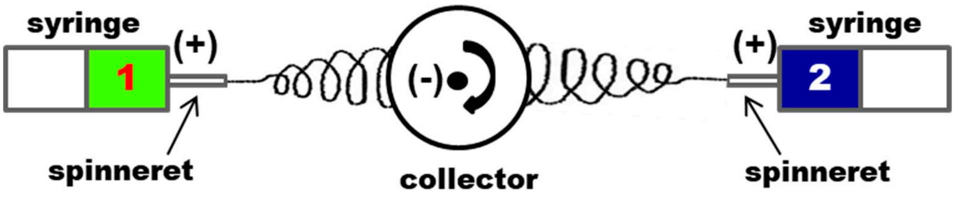

Scheme S1. Schematic illustration of the dual spinneret electrospinning setup used for preparation of (PLA + PVA/Ch/RA) and (PLA/LHC + PVA/Ch/RA) multifunctional fibrous materials: (1) PLA or PLA/LHC solution labeled with fluorescein; (2) PVA/Ch/RA solution.

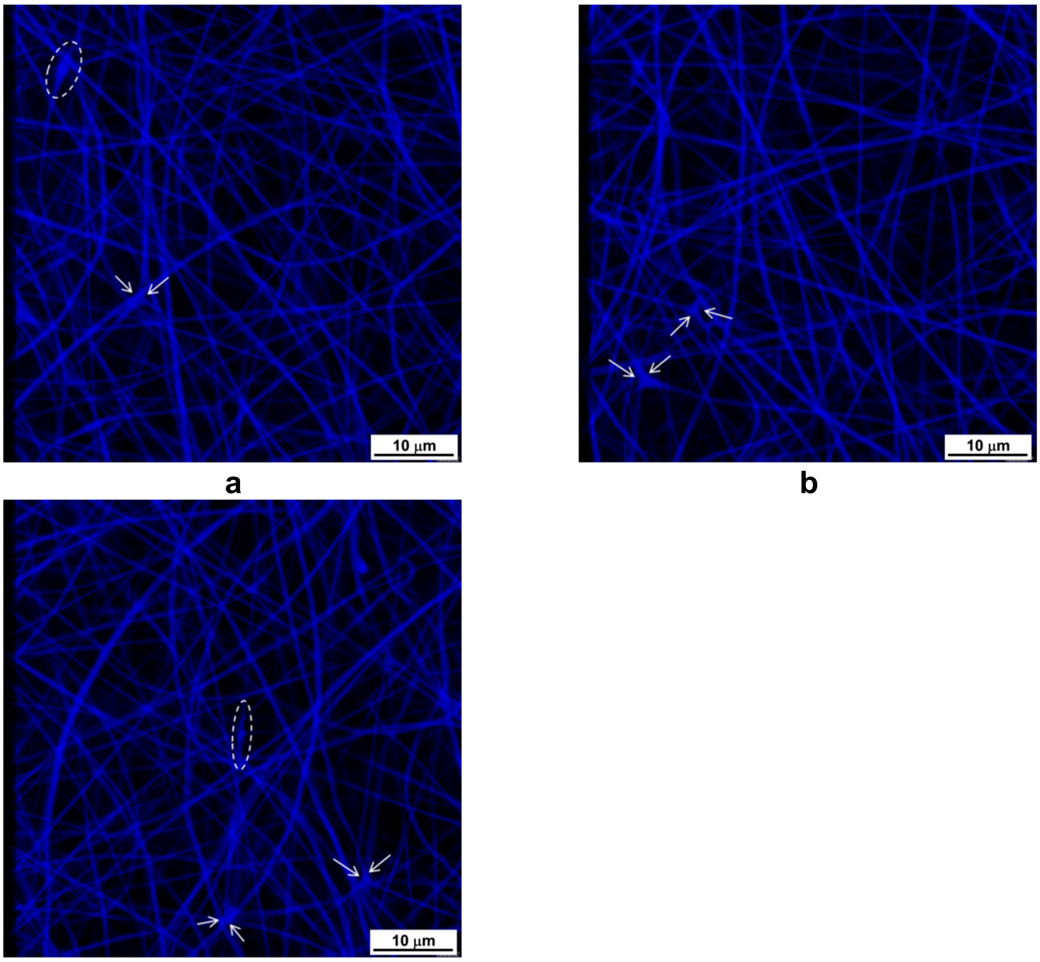

Figure S1. CLSM images of PVA/Ch/RA (10 wt%) fibers. PVA/Ch/RA fibers are stained in blue by the embedded RA. Selected images of Z-layers at a depth of 3 μm (a), 5 μm (b), and 10 μm (c). The Z step of the images is 1 μm. Scale bars = 10 μm. The branching of some of the main fibers is indicated with arrow, and the spindle-like defects are marked with dotted line.

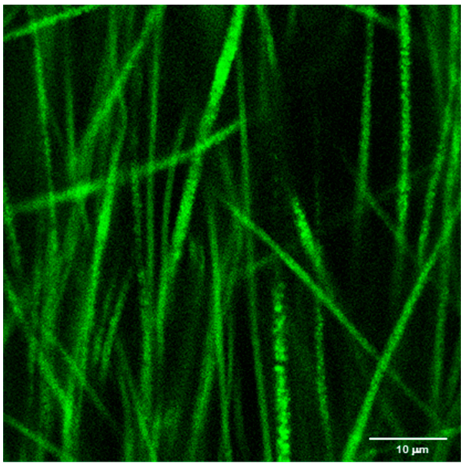

a

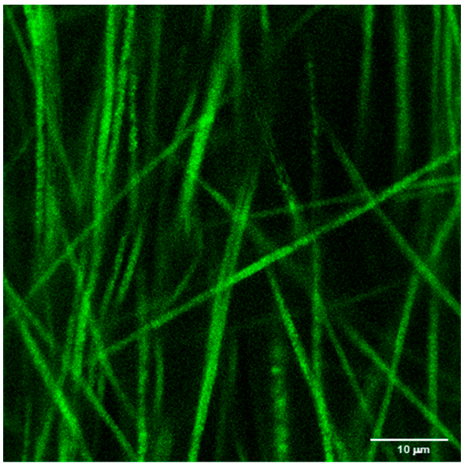

b

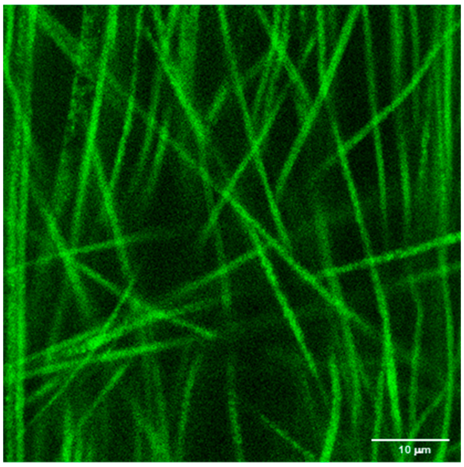

c

Figure S2. CLSM images of PLA/LHC/fluorescein (0.5 wt%) fibers. PLA/LHC/fluorescein fibers are stained in green by the embedded fluorescein. Selected images of Z-layers at a depth of 3 µm (a), 5 µm (b), and 9 µm (c). The Z step of the images is 1 µm. Scale bars = 10 µm.

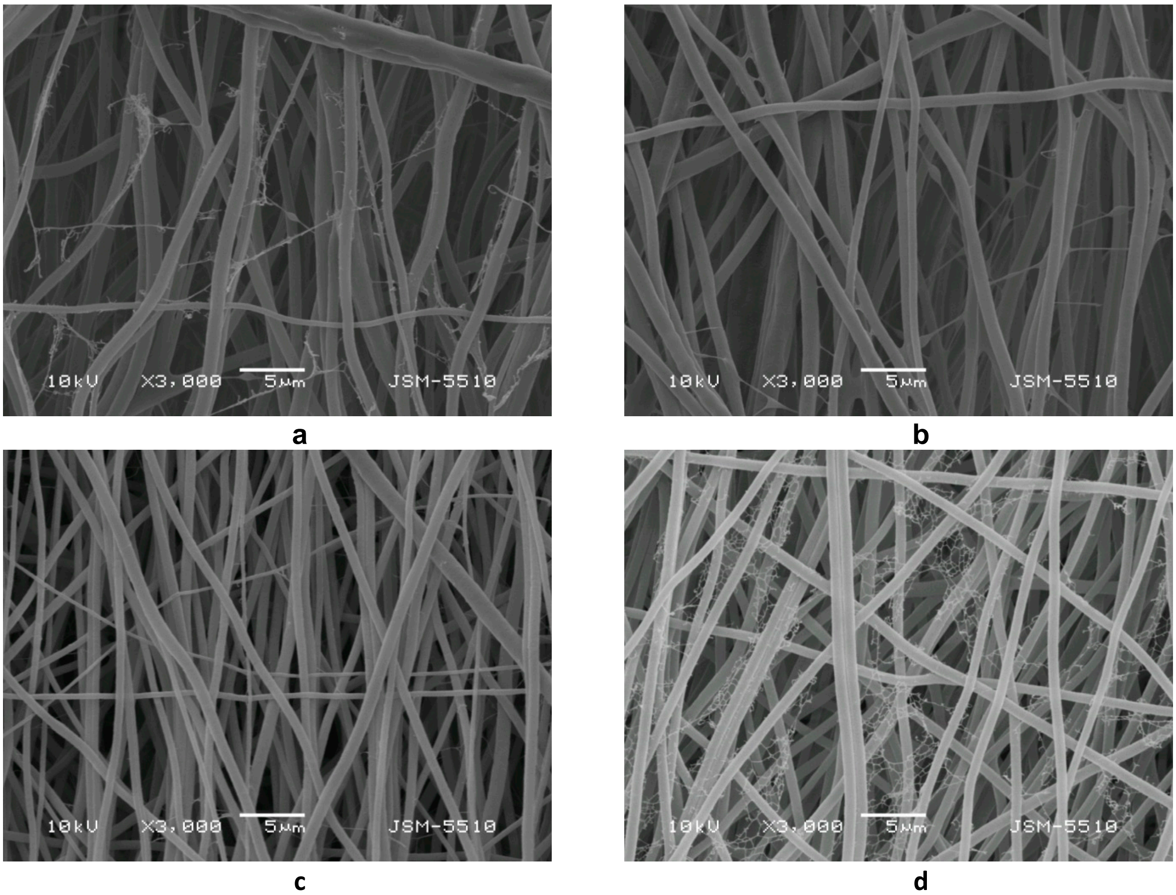

Figure S3. SEM micrographs of the fabricated electrospun mats: (PLA + PVA/Ch/RA) after 24 h (a) and 48 h (b) immersion in PBS buffer; and (PLA/LHC + PVA/Ch/RA) after 24 h (c) and 48 h (d) immersion in PBS buffer.

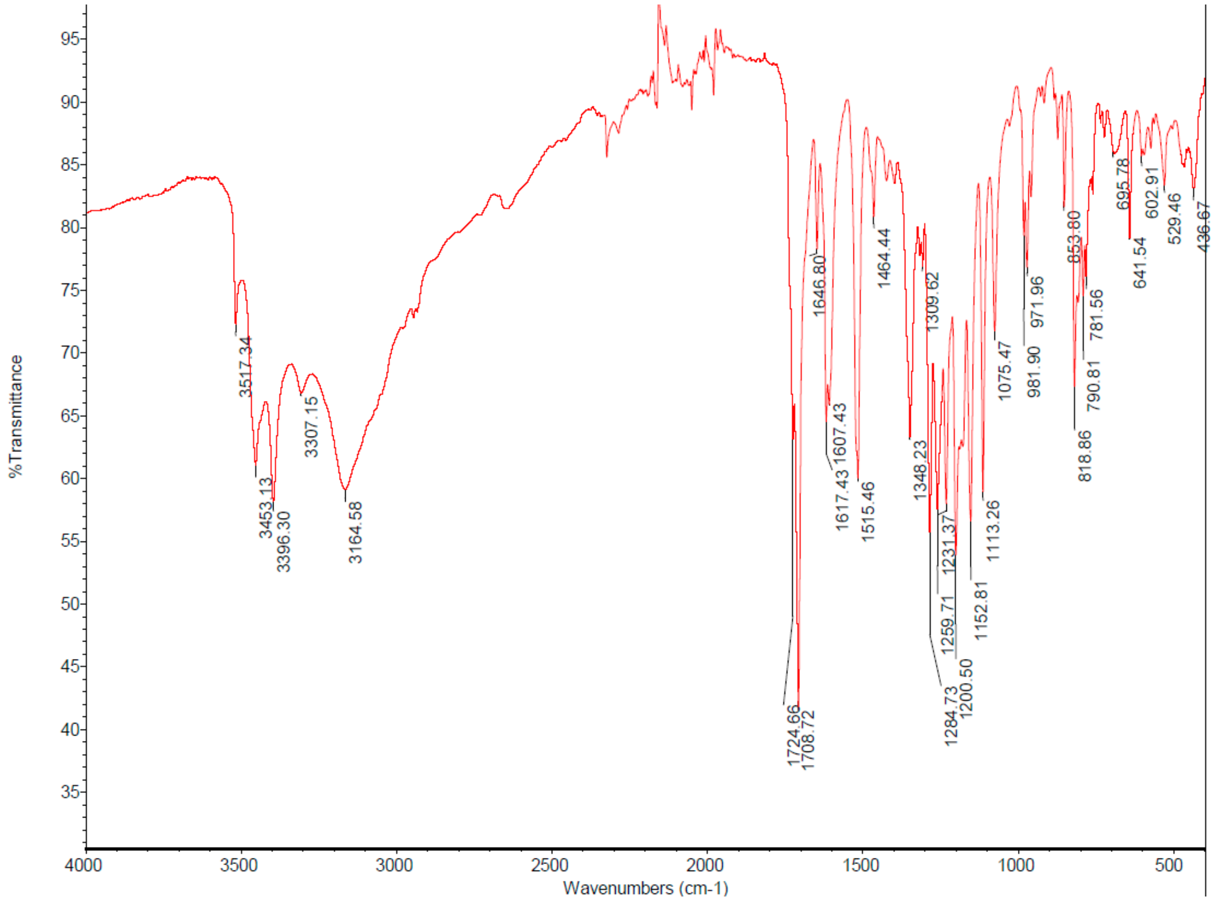

Figure S4. ATR–FTIR spectrum of RA (powder).

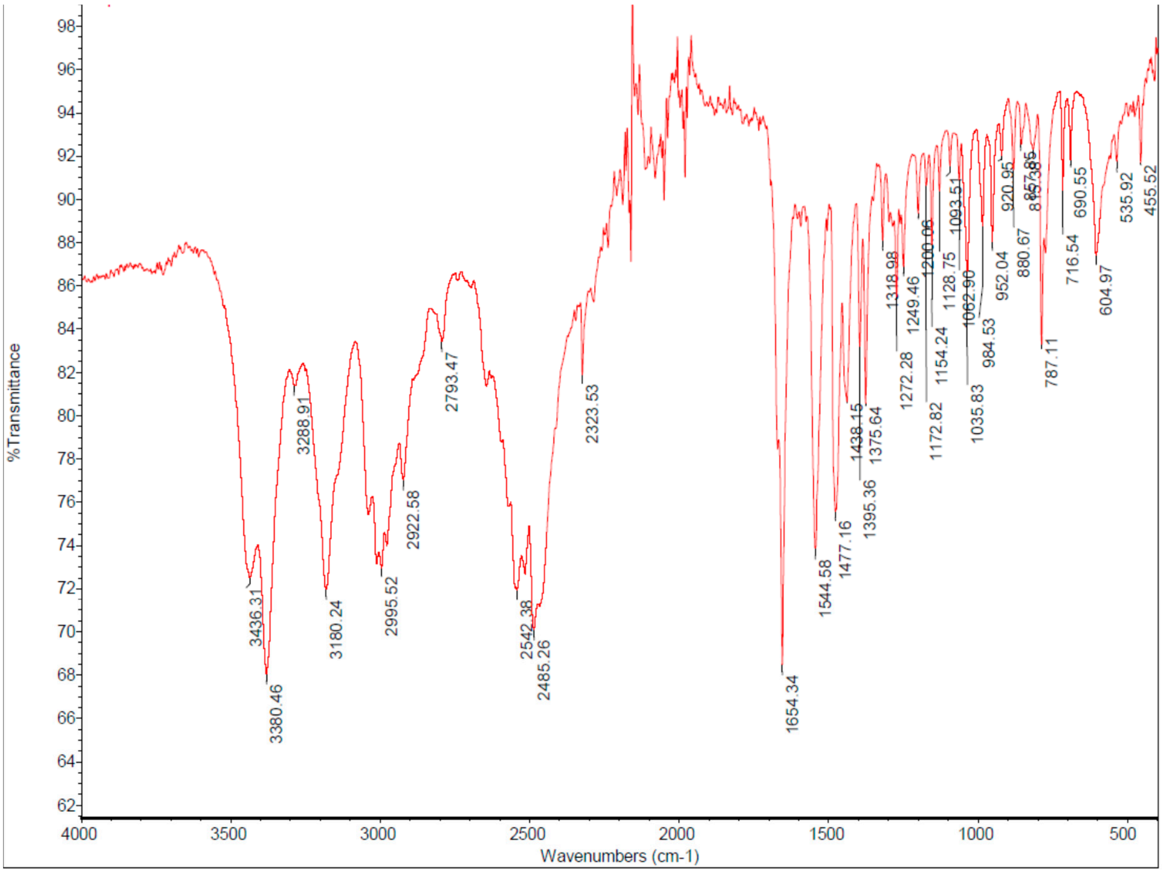

Figure S5. ATR–FTIR spectrum of LHC (powder).

Table S3. Thermal characteristics of the mats and degree of crystallinity of PVA

**Mats**	**T_m^{RA}, °C**	**T_g^{PVA}, °C**	**T_m^{PVA}, °C**	**ΔH_m^{PVA}, J/g**	**χ^{PVA}, % [(a)]**
PVA	-	42	205	59.4	42.8
PVA/Ch	-	29	204	48.9	37.0
PVA/Ch/RA	-	40	200	40.4	33.7

[(a)] PVA crystallinity degree in the fibrous materials was calculated using the following equation: χ_c^{PVA}, % = $\Delta H_m^{PVA}/(\Delta H_m^{PVA,0} \times W^{PVA}) \times 100$; $\Delta H_m^{PVA,0}$ = 138.6 J/g [39], and W^{PVA} was the weight fraction of PVA in the fibrous materials.

Table S4. Thermal characteristics of the mats and degree of crystallinity of PVA and PLA

**Mats**	**T_g^{PLA}, °C**	**T_{cc}^{PLA}, °C**	**ΔH_{cc}^{PLA}, J/g**	**T_m^{PLA}, °C**	**ΔH_m^{PLA}, J/g**	**χ^{PLA}, % [(a)]**	**T_g^{PVA}, °C**	**T_m^{PVA}, °C**	**ΔH_m^{PVA}, J/g**	**χ^{PVA}, % [(b)]**
PLA	57	78	25	165	51.8	29[(a)]	-	-	-	-
PLA/LHC	53	73	24.4	164	52.7	34[(b)]	-	-	-	-
PVA	-	-	-	-	-	-	42	205	59.4	43
PLA + PVA/Ch/RA	55	74	20.4	162	39.3	30[(c)]	38	200	13.4	33.5
PLA/LHC + PVA/Ch/RA	55	73	17.8	164	41.2	41[(e)]	41	200	13.4	33.5

[(a)]PLA crystallinity degree in the fibrous materials was calculated using the following equation: χ_c^{PLA}, % = $[(\Delta H_m^{PLA} - \Delta H_{cc}^{PLA})/(\Delta H_m^{PLA,0} \times W^{PLA})] \times 100$; $\Delta H_m^{PLA,0}$ = 93.0 J/g [38], and W^{PLA} was the weight fraction of PLA in the fibrous materials.

[(b)]PVA crystallinity degree in the fibrous materials was calculated using the following equation: χ_c^{PVA}, % = $[\Delta H_m^{PVA}/(\Delta H_m^{PVA,0} \times W^{PVA})] \times 100$; $\Delta H_m^{PVA,0}$ = 138.6 J/g [39], and W^{PVA} was the weight fraction of PVA in the fibrous materials.

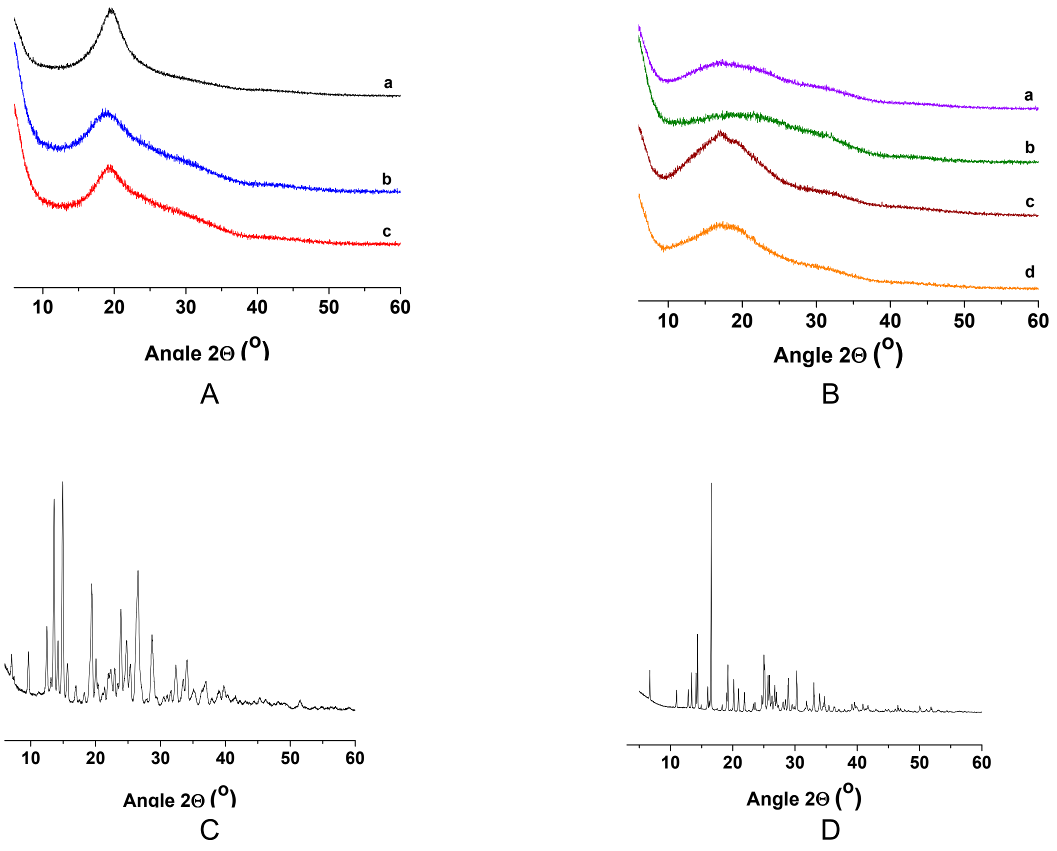

Figure S6. XRD patterns of: (A) (a) PVA, (b) PVA/Ch, (c) PVA/Ch/RA (10 wt%); (B) (a) PLA, (b) PLA/LHC, (c) (PLA + PVA/Ch/RA), (d) (PLA/LHC + PVA/Ch/RA), (C) RA powder, and (D) LHC powder.

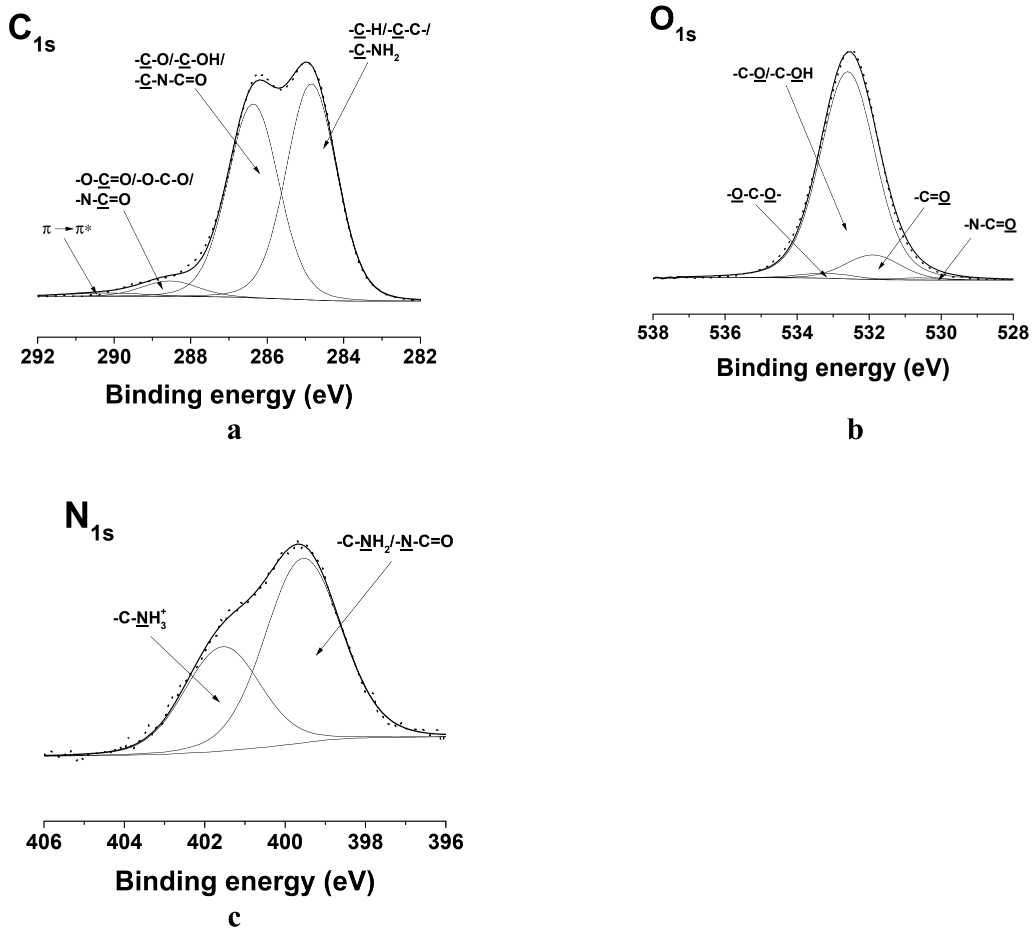

Figure S7. XPS peak fittings for PVA/Ch/RA (10wt% RA) mat [(a) C1s, (b) O1s, (c) N1s].

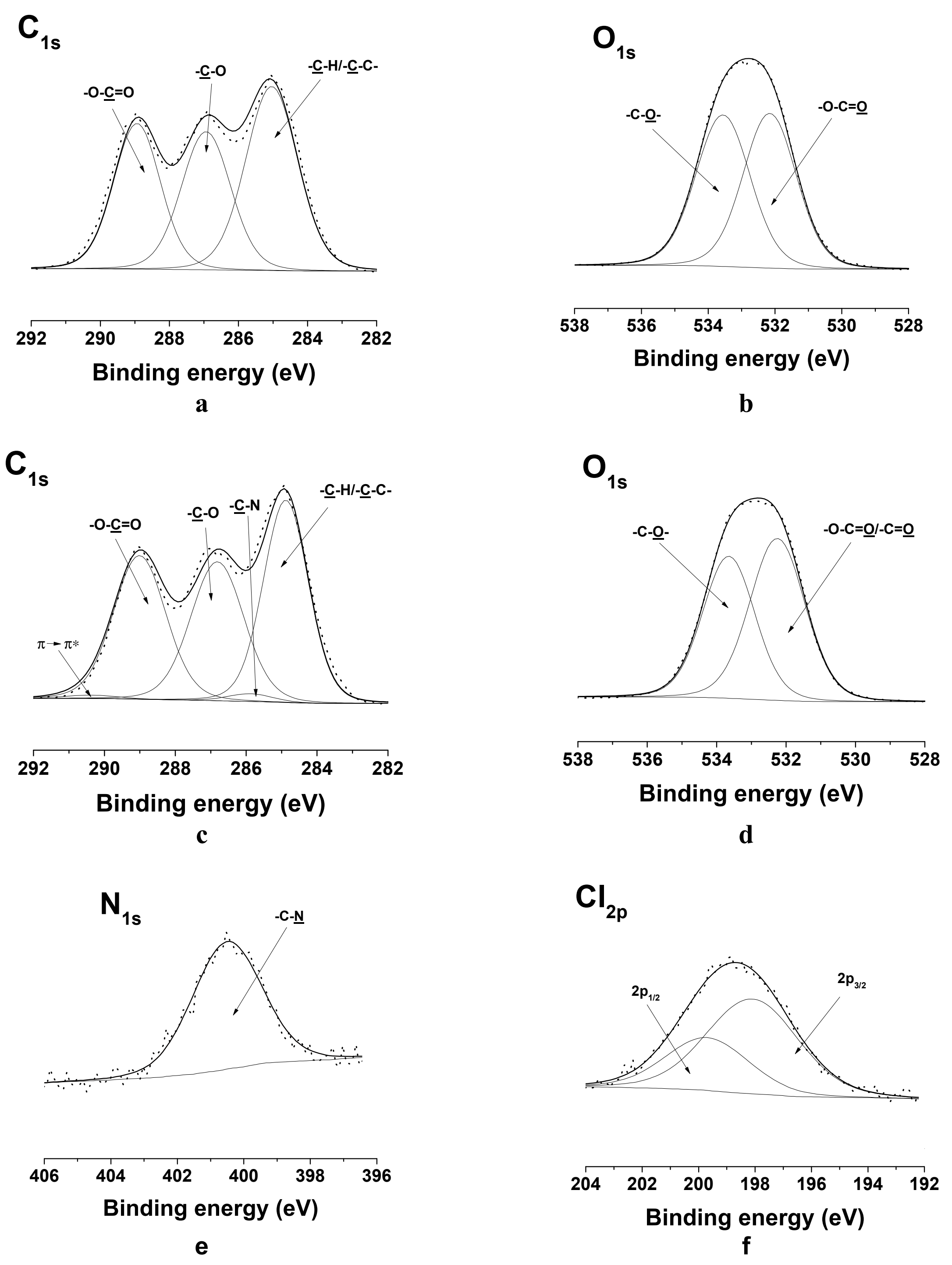

Figure S8. XPS peak fittings for PLA mat [(a) C1s, (b) O1s] and PLA/LHC mat [(c) C1s, (d) O1s, (e) N1s, (f) Cl2p].

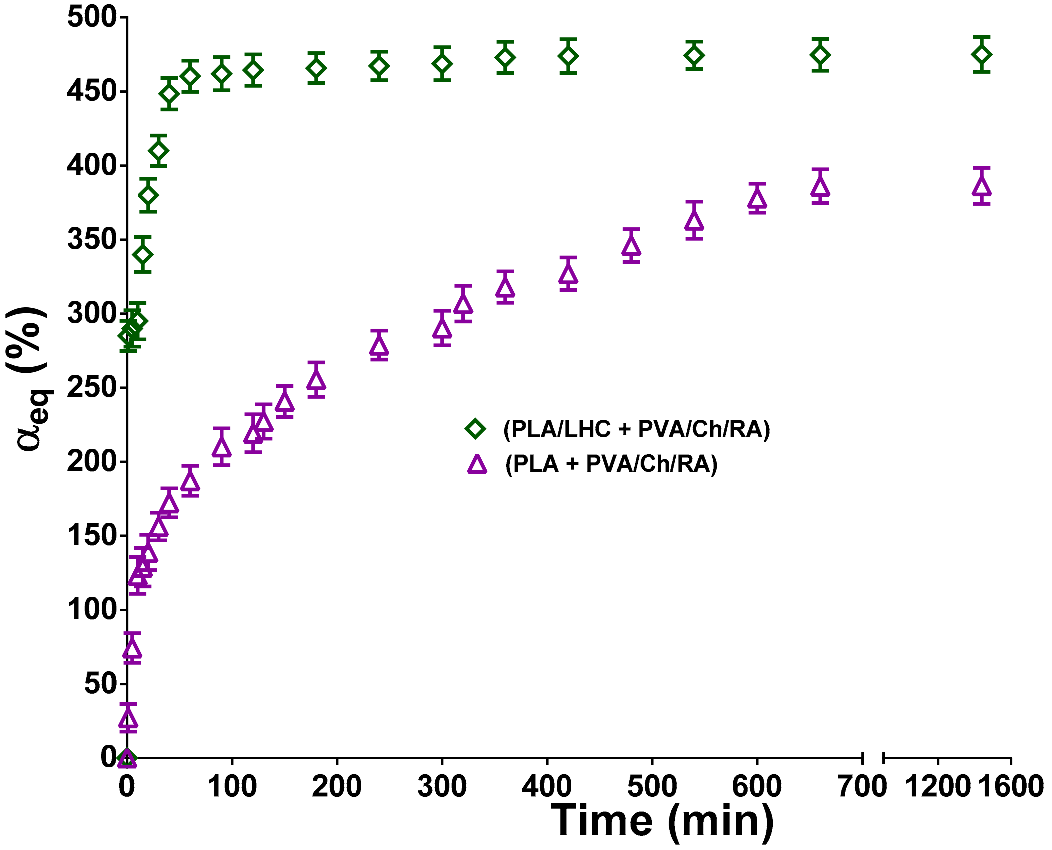

Figure S9. Equilibrium swelling degree (α_{eq}), of (PLA + PVA/Ch/RA) mat and of (PLA/LHC + PVA/Ch/RA) mat in PBS (pH 7.4) at 37 °C, versus time.

Figure S10. *In vitro* study of the LHC release from mats: PLA/LHC (10 wt% LHC) and (PLA/LHC (10 wt% LHC) + PVA/Ch/RA (10 wt% RA)); PBS, 37 °C, pH 7.4, ionic strength, 0.1.

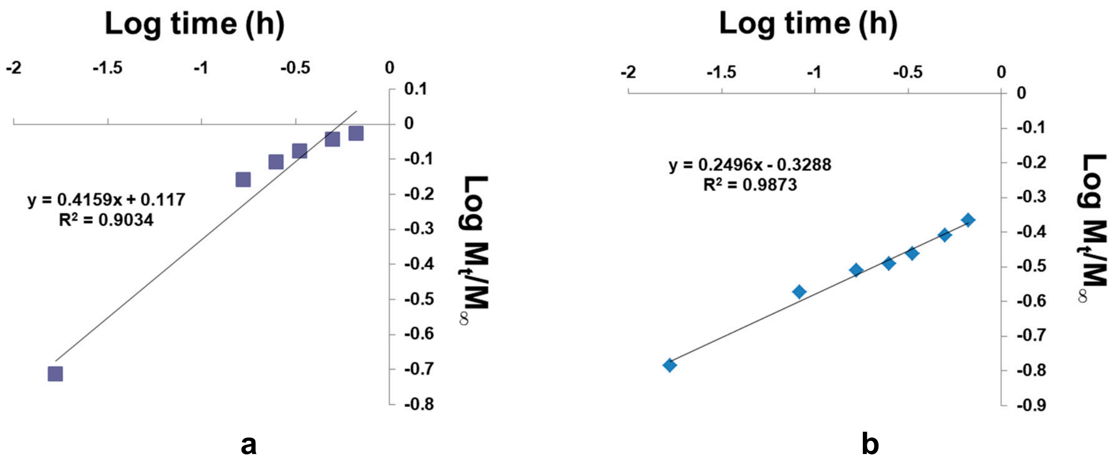

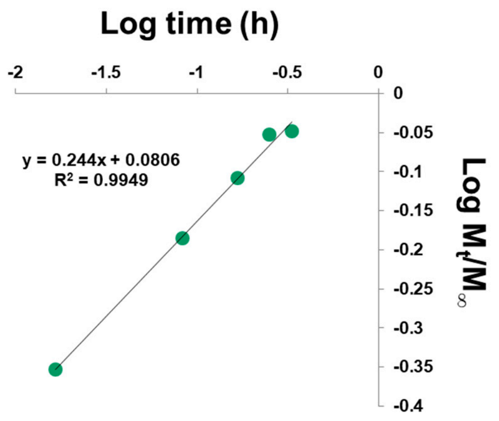

c

Figure S11. Korsmeyer-Peppas model of RA release from mats: PVA/Ch/RA (10 wt% RA) (a), (PLA + PVA/Ch/RA (10 wt% RA)) (b); and (PLA/LHC (10 wt% LHC) + PVA/Ch/RA (10 wt% RA)) (c) in PBS at 37ºC, pH 7.4, ionic strength 0.1.

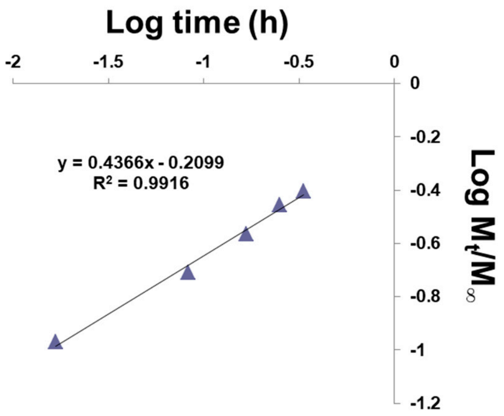

a

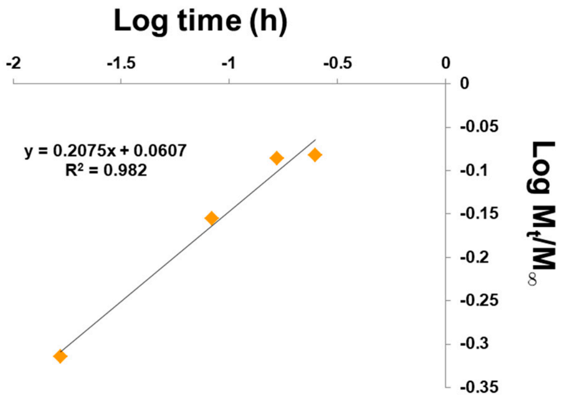

b

Figure S12. Korsmeyer-Peppas model of LHC release from mats: PLA/LHC (10 wt% LHC) (a) and (PLA/LHC (10 wt% LHC) + PVA/Ch/RA (10 wt% RA)) (b) in PBS at 37ºC, pH 7.4, ionic strength 0.1.

Table S5. Results from the One-way analysis of variance (ANOVA) utilized to assess the statistical significance of the data presented in Figure 12 of the manuscript using GraphPad PRISM.

Bonferroni's Multiple Comparison Test*	Mean Diff.	t	Significant? P < 0.05?	Summary	95% CI of diff.
1 vs 2	70.44	844.6	Yes	***	70.14 to 70.73
1 vs 3	90.76	1147	Yes	***	90.49 to 91.04
1 vs 4	90.98	1195	Yes	***	90.71 to 91.24
1 vs 5	82.07	984.1	Yes	***	81.78 to 82.36
1 vs 6	70.23	842.0	Yes	***	69.93 to 70.52
1 vs 7	2.255	28.50	Yes	***	1.977 to 2.533
1 vs 8	4.188	50.21	Yes	***	3.895 to 4.480
1 vs 9	-2.875	34.47	Yes	***	-3.168 to -2.582
2 vs 3	20.33	256.9	Yes	***	20.05 to 20.60
2 vs 4	20.54	269.8	Yes	***	20.27 to 20.80
2 vs 5	11.63	139.5	Yes	***	11.34 to 11.93
2 vs 6	-0.2125	2.548	No	ns**	-0.5054 to 0.08037
2 vs 7	-68.18	861.8	Yes	***	-68.46 to -67.90
2 vs 8	-66.25	794.4	Yes	***	-66.54 to -65.96
2 vs 9	-73.31	879.1	Yes	***	-73.61 to -73.02
3 vs 4	0.2120	2.968	No	ns**	-0.03880 to 0.4628
3 vs 5	-8.693	109.9	Yes	***	-8.971 to -8.415
3 vs 6	-20.54	259.6	Yes	***	-20.82 to -20.26
3 vs 7	-88.51	1187	Yes	***	-88.77 to -88.25
3 vs 8	-86.58	1094	Yes	***	-86.85 to -86.30
3 vs 9	-93.64	1184	Yes	***	-93.92 to -93.36
4 vs 5	-8.905	117.0	Yes	***	-9.172 to -8.638
4 vs 6	-20.75	272.6	Yes	***	-21.02 to -20.48
4 vs 7	-88.72	1242	Yes	***	-88.97 to -88.47
4 vs 8	-86.79	1140	Yes	***	-87.05 to -86.52
4 vs 9	-93.85	1233	Yes	***	-94.12 to -93.58
5 vs 6	-11.85	142.0	Yes	***	-12.14 to -11.55
5 vs 7	-79.82	1009	Yes	***	-80.09 to -79.54
5 vs 8	-77.88	933.9	Yes	***	-78.18 to -77.59
5 vs 9	-84.95	1019	Yes	***	-85.24 to -84.65
6 vs 7	-67.97	859.1	Yes	***	-68.25 to -67.69
6 vs 8	-66.04	791.8	Yes	***	-66.33 to -65.74
6 vs 9	-73.10	876.5	Yes	***	-73.39 to -72.81

7 vs 8	1.932	24.43	Yes	***	1.655 to 2.210
7 vs 9	-5.130	64.84	Yes	***	-5.408 to -4.852
8 vs 9	-7.063	84.68	Yes	***	-7.355 to -6.770

* 1- RA solution, 2- LHC solution, 3- PLA mat, 4- PVA mat, 5- PVA/Ch mat, 6- PLA/LHC mat, 7- PVA/Ch/RA mat, 8- (PLA + PVA/Ch/RA) mat, 9- (PLA/LHC + PVA/Ch/RA) mat, **ns - not significant

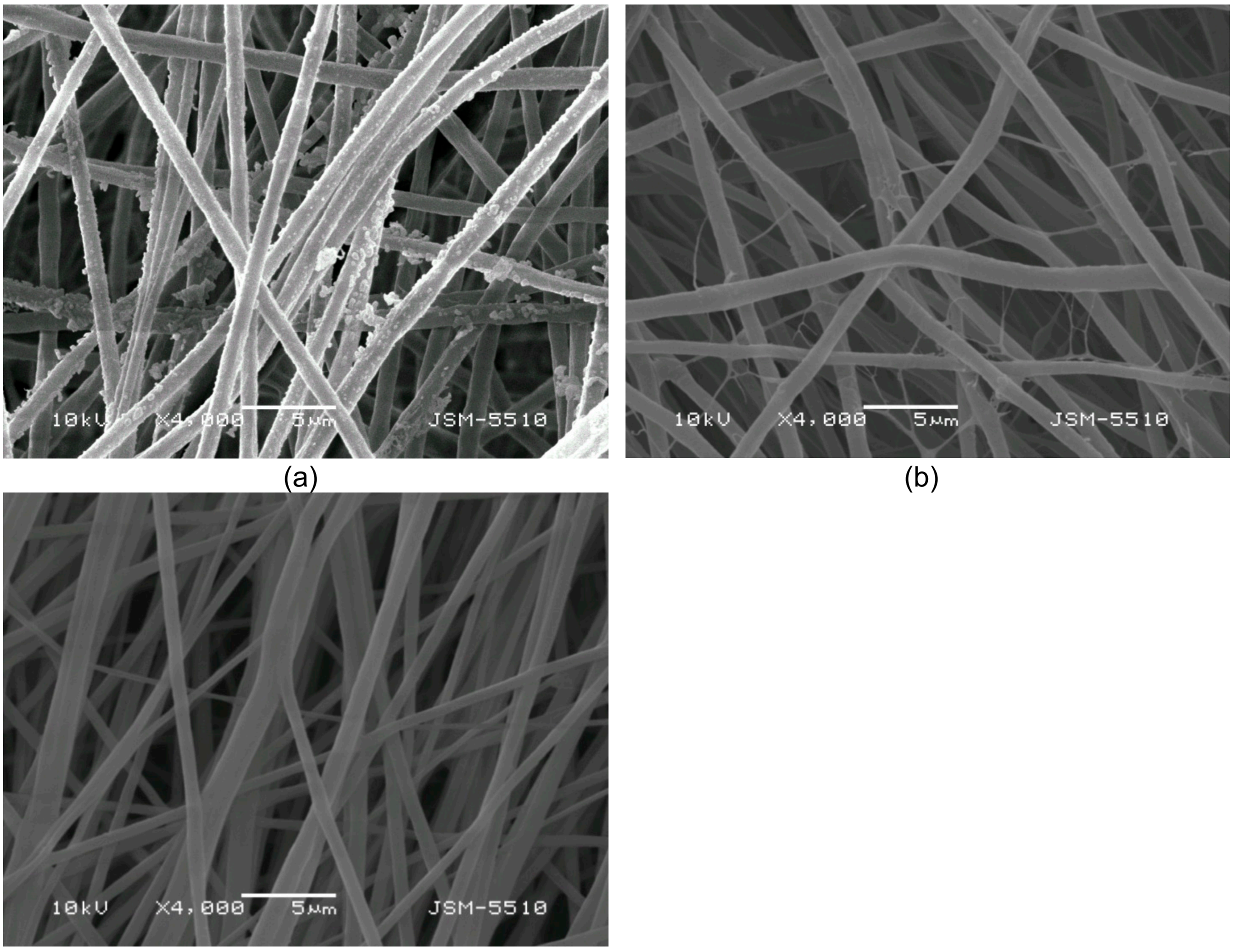

(a) (b)

(c)

Figure S13. SEM micrographs of fibrous materials that have been incubated in *S. aureus* cell culture (10^7 cells/mL) for 24 h at 37°C, (a) PLA, (b) (PLA + PVA/Ch/RA) and (c) (PLA/LHC + PVA/Ch/RA); magnification ×4000.
